# Mechanistic signatures of comorbid PTSD with cognitive impairment implicate cortisol-induced neural toxicity

**DOI:** 10.1038/s41386-026-02358-6

**Published:** 2026-02-12

**Authors:** Zeming Kuang, Anthony G. Chesebro, Sophia-Gisela Strey, Sean A. P. Clouston, Benjamin J. Luft, Lilianne R. Mujica-Parodi

**Affiliations:** 1 Department of Biomedical Engineering, State University of New York at Stony Brook, Stony Brook, NY USA; 2 Department of Radiology, Athinoula A. Martinos Center for Biomedical Imaging, Massachusetts General Hospital and Harvard Medical School, Charlestown, MA USA; 3 Laufer Center for Physical and Quantitative Biology, Stony Brook University, Stony Brook, NY USA; 4 Program in Public Health, Renaissance School of Medicine at Stony Brook University, Stony Brook, NY USA; 5 Department of Family, Population, and Preventive Medicine, Renaissance School of Medicine at Stony Brook, Stony Brook, NY USA; 6 World Trade Center Health Program, Commack, NY USA; 7 Department of Medicine, Renaissance School of Medicine at Stony Brook University, Stony Brook, NY USA; 8 Santa Fe Institute, Santa Fe, NM USA

**Keywords:** Post-traumatic stress disorder, Predictive markers

## Abstract

The men and women who worked in rescue and recovery operations at the 9/11 World Trade Center site are developing cognitive impairment (CI) at mid-life, decades before CI is usually detected. To date, one of the most consistent risk factors for CI in this population is symptoms of post-traumatic stress disorder (PTSD). However, little is known about the mechanistic cascade that drives stress-related neurological changes to accelerate cognitive decline in the human brain. We used machine learning to identify distinct brain signatures from functional magnetic resonance imaging between trauma-exposed healthy controls (TEHC; *N* = 30; 21 men), PTSD without CI (PTSD-CI; *N* = 19; 16 men), and PTSD with CI (PTSD + CI; *N* = 22; 18 men). We compared the spatial gradient of each functional signature to the distribution of mRNA expression in the brain. We applied structural equation modeling (SEM) to infer mechanistic cascades specific to each group. While modest accuracy was achieved for the PTSD–CI versus TEHC signature (0.67), clear differentiation was observed for PTSD + CI versus TEHC (0.73) and PTSD + CI versus PTSD–CI (0.85). Consistent significant correlations were found between PTSD + CI signatures and *ZNF48*, *TOMM40*, and *GRIN1* expression distributions. The cortisol-induced neurotoxicity pathway was consistently found with the PTSD + CI signature, while the p53 signaling pathway was observed across all PTSD signatures. Our results reinforce peripheral biomarkers from a previous transcriptomic study and suggest functional biomarkers in PTSD and PTSD-related CI. Furthermore, our SEM results suggest that PTSD and PTSD-related CI may diverge at the mechanistic level, with neurotoxicity being specific to CI.

## Introduction

Personnel involved in rescue and recovery operations at the 9/11 World Trade Center (WTC) site are developing cognitive impairment (CI) at mid-life [1–4], decades earlier than typically detected [5]. Two consistent risk factors for cognitive dysfunction and impairment in this population include long-term exposure to the WTC disaster sites and symptom severity of post-traumatic stress disorder (PTSD) [3, 4, 6, 7]. This correlation between PTSD severity and accelerated CI is consistent across multiple studies with various trauma sources [6, 8–10]. Unlike CI due to brain aging, CI in PTSD is more efficiently explained by PTSD severity than neurodegeneration biomarkers such as β-amyloid [9]. Collectively, this evidence indicates that shared biological mechanisms may contribute to both PTSD and CI [6, 9, 10]. Among the most consistent candidates are dysregulated stress response pathways [11–13] and apoptosis-related processes [14, 15]. Both have been consistently shown to be altered in PTSD and, when disrupted, can have profound consequences for brain function [16, 17].

The relationship between stress, allostatic load, and impaired cognition has been well-established in both human and animal models [18]. Chronic stress elevates allostatic load, resulting in prolonged glucocorticoid exposure that induces neurotoxicity in a spatially heterogeneous manner, with the prefrontal cortex and hippocampus being particularly vulnerable [18–20]. Elevated glucocorticoid levels, often measured through cortisol, have been linked to a greater risk of cognitive decline and Alzheimer’s disease [19] and PTSD [21]. Typically released in response to a perceived threat, cortisol regulation can become disrupted in individuals with PTSD [17, 21]. Research suggests that PTSD is associated with lower baseline cortisol levels, yet an exaggerated cortisol response to stress [21]. This dysregulation may contribute to hypervigilance, heightened startle response, and difficulty in processing traumatic memories [22]. Additionally, altered cortisol patterns may impair fear extinction processes, which are crucial for recovery from trauma [23], and may contribute to the accelerated cognitive decline observed in PTSD. Consistent with these neuroendocrine alterations, transcriptomic analyses in a WTC first responder PTSD cohort identified the glucocorticoid receptor signaling pathway as the top differentially expressed pathway [24].

An alternative explanation for accelerated cognitive impairment (CI) in PTSD involves dysregulated oxidative stress [25]. While moderate oxidative stress can serve adaptive roles, excessive levels are neurotoxic [26]. This imbalance is partly reflected in the B-cell lymphoma 2 (BCL2) to Bcl-2-associated X protein (BAX) ratio, which is altered in the hippocampus and medial prefrontal cortex of PTSD animal models [27–29], as well as at both genetic and protein levels in PTSD veterans [14]. Mitochondrial dysfunction, another hallmark of PTSD [30], further amplifies oxidative stress and promotes neuroinflammation. Importantly, both the BCL2/BAX pathway and mitochondrial apoptosis converge on p53, which has been implicated in a reactive oxygen species (ROS)/JNK/p53 cascade in PTSD animal models [31]. Given that p53-related markers are also linked to cognitive impairment [32–34], dysregulation of this pathway may represent a mechanistic bridge between PTSD and CI.

While deficits in specific cognitive domains have been explored in functional brain studies of PTSD [35–37], the biological connections between PTSD and accelerated cognitive decline remain unestablished in the human brain. Biomarkers supporting the two hypothesized theories have been observed in blood samples [14, 24] and well-studied in animal/cell models [18, 20, 27–29, 38]. However, these findings rely on the assumption that results from peripheral tissues and model systems translate directly to the human brain. It is not yet clear whether cortisol dysregulation, p53-driven apoptosis, and cognitive impairment in PTSD are mechanistically related or whether they represent parallel outcomes of PTSD as a common upstream cause. A further challenge is that most mechanistic research has relied on animal and cell models focused on brain-level changes [18, 20, 27–29, 38]. By contrast, human studies have primarily shown correlations between PTSD severity and cognitive decline [6, 8–10], alongside alterations in stress response and genetic/proteomic profiles in blood [11–13, 24, 39]. Advances in whole-brain mRNA expression maps now provide an opportunity to mechanistically connect neural alterations and genetic and proteomic effects in human subjects.

**Fig. 1:**
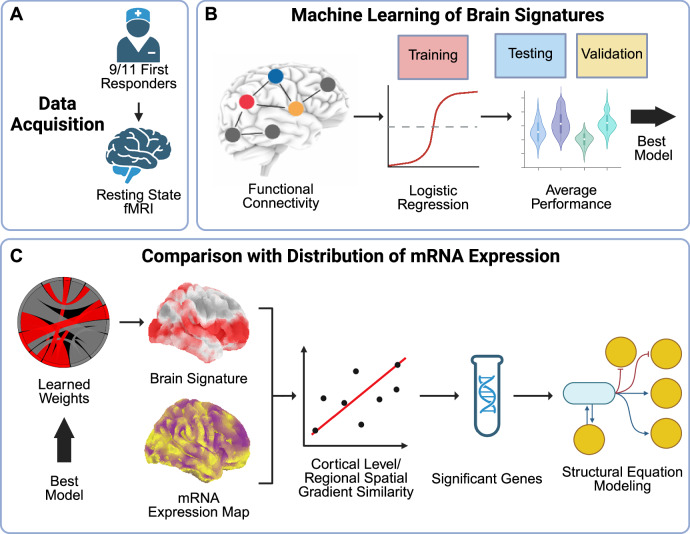
Schematic of the analytic pipeline. **A** Resting-state functional MRI scans were obtained from 9/11 first responders classified into TEHC, PTSD + CI, and PTSD–CI groups. **B** Functional connectivity matrices derived from group-level IC pairs were used to train machine learning models, and the best-performing model was selected for further analysis. **C** The learned weights from the best-performing model were projected back into brain space to derive phenotype-specific brain signatures. Spatial gradient similarity between each signature and mRNA expression maps was quantified using Spearman’s R and modeled with SEM to infer potential molecular pathways.

Here, we address this gap by dissociating stress and cognitive effects in PTSD using machine learning (Fig. 1B) [40]. We compare functional neurological signatures across three groups: individuals with PTSD with CI (PTSD + CI), individuals with PTSD without CI (PTSD–CI), and trauma-exposed healthy controls who did not go on to develop either PTSD or CI (TEHC). Learned brain signatures were compared with the mRNA expression distribution across the human brain of genes that are differentially expressed in PTSD peripheral studies or involved in PTSD-related biological processes (Fig. 1C). Statistical relationships between brain signatures and gene distributions were evaluated in the context of the two competing PTSD hypotheses: cortisol-induced neurotoxicity and p53 apoptosis. These hypotheses allowed us to infer sensitivity to candidate biological pathways underlying functional brain changes specific to PTSD and a clinical trajectory with the subsequent development of CI. Importantly, our study leverages one of the few well-characterized cohorts of WTC responders, a highly trained, healthy workforce exposed to a single, severe traumatic event without concomitant head injury, distinct from typical PTSD samples that often include chronic trauma or co-occurring TBI. By further distinguishing PTSD participants with and without cognitive impairment, our design enables the identification of neural mechanisms specific to PTSD as well as those associated with PTSD-related cognitive decline, providing a rare and robust framework for bridging peripheral biomarkers with neural phenotypes and clinical outcomes.

## Patients and methods

### Participants and clinical assessment

Data collection was reviewed and approved by the IRB at Stony Brook for this study (CORIHS-A). All standards were met, and study requirements were fulfilled. All participants provided informed, written consent to collect and store neuroimages for research purposes. Our cohort included *N* = 71 WTC responders (55 men [77%]; mean [SD] age, 55.7 [5.03] years) who underwent T1-weighted (T1w) structural and functional magnetic resonance imaging (fMRI; Fig. 1A) between July 2017 and December 2019 as part of the WTC Imaging Archive at the Stony Brook University WTC Health Program on Long Island, NY. Eligibility was certified by the Centers for Disease Control based on screening and documentation confirming work at the WTC during the specified period and duration. PTSD and dementia diagnoses were assessed using the Structured Clinical Interview for DSM-IV (SCID) [41] and Montreal Cognitive Assessment, respectively. Lifetime trajectories of PTSD symptoms were assessed in the diagnostic screening. Specifically, the PTSD Checklist (PCL) was completed by participants annually since 2002, and only individuals who had 5–10 PCL assessments with >50% scores exceeding 44 and an average score exceeding 44 were considered to have consistently high symptoms. ANOVA found no PTSD symptom severity (from SCID) difference between PTSD + CI and PTSD-CI (all *p* > 0.05). At the same time, there was a significant symptom severity difference between the non-PTSD and PTSD groups (all *p* < 0.001). Only individuals with consistently high PTSD symptoms that corroborated the diagnosis at the screening were included in the PTSD cohorts. Mild early-onset dementia was diagnosed if responders had a history of objective cognitive decline and evidence of objective CI on two tests in one domain, with scores falling <1.5 SD of normative data on the CogState, while still functioning independently. Dementia diagnosis followed NIA-AA guidelines, requiring criteria for mild early-onset dementia plus a second domain of impairment, i.e., two tests in two domains <1.5 SD on the CogState, with impaired daily living activities. Participants with mild early-onset dementia or dementia without PTSD were excluded from the analysis. Because it is unclear whether CI in PTSD is a unique or progressive disorder, CI was used to cover the broader concept instead of dementia. To account for age-related cognitive changes, the PTSD-CI and TEHC groups were age-matched with the PTSD + CI cohort, which developed CI in their mid-fifties [2]. The majority of the participants were right-hand dominant, with 9% left-handed and 5% ambidextrous. Exclusion criteria include a history of psychosis, history of diagnosed neurological conditions, including diagnosed ADRD, other dementias, major stroke, multiple sclerosis, Parkinson’s disease; any head injury during the WTC or a history of military head trauma, including combustive blasts; current liver disease; current use of cognitively active medications; and undergoing active treatment like chemotherapy. The cohort was divided into three subgroups: PTSD–CI (*N* = 19, 16 men [84%]; mean [SD] age, 54.4 [5.06] years), PTSD + CI (*N* = 22, 18 men [82%]; mean [SD] age, 56.5 [5.34] years), and TEHC (*N* = 30, 21 men [70%]; mean [SD] age, 56.0 [4.79] years).

### MRI acquisition

T1w images and fMRI were acquired on a high-resolution 3-Tesla SIEMENS mMR Biograph scanner using a 20-channel head and neck coil. MRI acquisitions were performed a median of 22 days (mean [SD]: 26 [17.6] days) after clinical assessment. A high-resolution structural image was obtained using a magnetization-prepared rapid acquisition gradient echo (MPRAGE) sequence with parameters TR = 1900 ms, TE = 2.49 ms, TI = 900 ms, flip angle = 9 degrees, acquisition matrix = 256 × 256, and 224 slices, resulting in a voxel size of 0.89 × 0.89 × 0.89 mm. A 10-minute resting-state fMRI scan was performed using a continuous gradient echo-echo planar imaging (GE-EPI) sequence with TR = 1500 ms, TE = 27 ms, pulse angle = 80 degrees, field of view = 22 cm, acquisition matrix = 74 × 74, and slice thickness of 2.5 mm. During resting-state scans, participants were instructed to remain still, keep their eyes open, and avoid specific thoughts.

### MRI preprocessing

For MRI images, subject-level T1w and fMRI images were preprocessed using fMRIPrep (https://fmriprep.org/), version 20.2.3 [42]. Confound removal, bandpass filtering (0.01 Hz to 0.1 Hz), detrending, and standardization were performed using Nilearn (https://nilearn.github.io/). Framewise displacement (FD) was computed for each scan. Scans with a mean FD > 0.5 mm were excluded from analysis. White matter, CSF, and motion parameters (three translations and three rotations) were included as confound regressors. The dimensions of fMRI data were reduced into 25 dimensions using Multivariate Exploratory Linear Optimized Decomposition into Independent Components (MELODIC) in FSL (https://fsl.fmrib.ox.ac.uk/fsl/fslwiki/MELODIC/) [43]. Non-brain independent components (ICs) were manually selected and removed. Single-subject fMRI data were mapped to each IC dimension using dual regression (https://fsl.fmrib.ox.ac.uk/fsl/fslwiki/DualRegression/) [44]. Functional connectivity (FC) between each IC pair was computed using Nilearn. A total of 25 ICs were identified from the whole cohort (Figs. S4–S28). Among them, ICs 4, 16, 18, 20, and 21 appeared to reflect edge artifacts. IC 15 corresponded to white matter. IC 24 encompassed most subcortical regions but was excluded, as the primary focus of this study was on the neocortex, given the resolution. 16 ICs were kept for downstream analyses.

### Machine learning to identify functional brain signatures

Logistic regression models were trained using FCs between IC pairs as features to classify (1) PTSD + CI compared to TEHC, (2) PTSD–CI compared to TEHC, and (3) PTSD + CI compared to PTSD-CI (PTSDΔCI). An elastic net was applied to regularize overfitting. Hyperparameter tuning was performed on the regularization strength (C) and the L1-to-L2 (L1/L2) ratio using cross-validated grid search in scikit-learn (https://scikit-learn.org/). Model performance was evaluated across k-fold in leave-one-out cross-validation (LOOCV). K was chosen to be 7 to maximize training size while keeping two to three subjects for evaluation. Performance metrics used were accuracy and receiver operating characteristic area under curve (ROC-AUC) for standard performance assessment, and balanced accuracy and precision-recall area under curve (PR-AUC) to account for class imbalance. Models with and without class weight balancing were fitted and evaluated on the test set to mitigate the risk of overfitting, considering the additional variance introduced by balancing. Each IC was weighted by the summed weights of its associated FC from the averaged models across k-fold validation runs. The weighted ICs were combined to classify each class’s functional brain signature map.

### Spatial gradient similarity with mRNA expression maps

Microarray data from the Allen Human Brain Atlas (https://portal.brain-map.org/) provided spatial mRNA expression information across the brain. We used whole-brain mRNA expression maps by smoothing microarray data using variogram fitting [45]. We computed the correlation between the spatial gradients of functional brain signatures and the distribution of mRNA gene expression at both the cortical and smaller regional levels as defined by the AAL atlas [46]. Specifically, correlations were calculated across voxels within each AAL-labeled region after resampling the gene expression and functional maps into the same space. Cortical and subcortical level mRNA expression maps were generated separately and scaled differently [45], which did not permit whole-brain level comparison. Spearman’s rank correlation was used to capture nonlinear trends and reduce sensitivity to outliers. Permutation tests were conducted using BrainSMASH [47] to establish conservative statistical expectations for correlation, preserving spatial autocorrelation in randomly permuted brain maps. We generated 1000 permutation maps for each signature map and compared them to individual gene expression maps to derive null distributions of the expected correlation. P-values were estimated using a z-score approximation. Statistical corrections included the Benjamini-Hochberg (BH) Procedure for controlling the false discovery rate (FDR) and the Bonferroni Correction for controlling the family-wise error rate (FWER), both set at an α level of 0.05.

### Candidate PTSD-related genes and pathways

We conducted analyses with 168 genes, selected as significant genes and pathways derived from (1) human PTSD transcriptomic studies and (2) animal PTSD models, selected based on our two hypotheses (see Table S1 for details on individual gene selection). Cortisol-induced neurotoxicity included 13 genes related to cortisol activation [48, 49], NADPH production [50], N-methyl-D-aspartate receptors (NMDAR), and ERK1/2 genes, which potentially act downstream following NMDAR function alteration [38]. Seventeen genes related to the GC receptor were included to address additional effects for cortisol [24, 51–54]. Nine genes related to brain glucose metabolism were included for NADPH and glutamate production [54]. Twenty-eight genes downstream of p53 (KEGG, hsa04115) [55], which are involved in IGF-1/mTOR inhibition, apoptosis, and mitochondrial apoptosis, were also incorporated into the ROS/JNK/p53 apoptosis hypothesis [31]. To represent spatial vulnerability to ROS, nine antioxidant genes were included [56]. Alongside these hypotheses, we included 82 genes differentially expressed in blood in the WTC PTSD cohort [24] for data-driven profiling, as well as eight related to β-amyloid and tau production [1], which were examined in the WTC responder cohort.

### Structural equation modeling

**Fig. 2:**
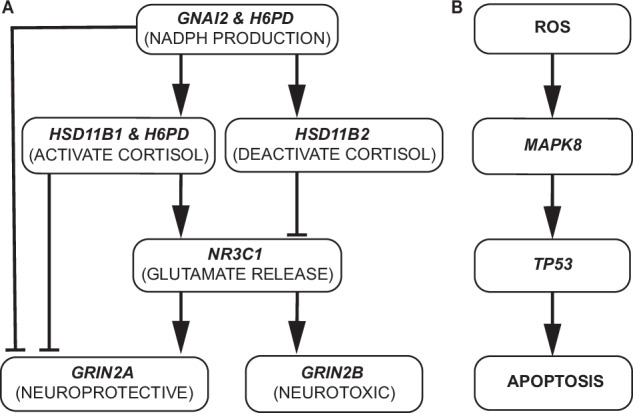
Two distinct yet interacting candidate biological pathways have been proposed for PTSD. **A** Under dysregulated stress responses, an excessive cortisol concentration modulates glutamatergic signaling, shifting from a dual role of neuroprotection and neurotoxicity to predominantly neurotoxic effects. This cortisol-induced neurotoxicity is proposed to contribute to neurodegeneration, leading to accelerated cognitive impairment in individuals with PTSD. **B** In the context of PTSD in general, blood biomarkers such as BCL2 to BAX ratio within the p53 apoptosis pathway are found across multiple animal and human studies [14, 29]. In an animal model study, the ROS/JNK/p53 signaling cascade is proposed to contribute to abnormal mental responses associated with PTSD [31].

Spatial gradient similarities between each signature and gene combination at regional levels were analyzed using structural equation modeling (SEM) to infer potential gene cascades. The semopy package (https://semopy.com/) was utilized. Similarity scores with a p-value less than 0.5 were retained to ensure adequate similarity to functional brain signatures while keeping enough data for the fit. Connections in the hypothesized pathways (see Fig. 2) were reversed, and gene hierarchies were permuted. Fit quality was evaluated using three indices: comparative fit index (CFI > 0.95), Tucker-Lewis index (TLI > 0.95), and root mean square error of approximation (RMSEA < 0.05). More lenient cutoffs were considered acceptable for comparison purposes.

## Results

### Functional brain signatures

Functionally related but distinct brain signatures were identified between PTSD ± CI. Model performance was evaluated using 7-fold LOOCV, and reported metrics reflect mean [standard deviation] across folds. The best-performing model for PTSD + CI was obtained with an L1/L2 ratio of 1 and *C* = 1 (accuracy = 0.73 [0.09]; balanced accuracy = 0.72 [0.11]; ROC-AUC = 0.85 [0.11]; PR-AUC = 0.74 [0.23]). For PTSDΔCI, the optimal model used an L1/L2 ratio of 1 and *C* = 21 (accuracy = 0.85 [0.12]; balanced accuracy = 0.86 [0.12]; ROC-AUC = 0.89 [0.17]; PR-AUC = 0.88 [0.20]). For PTSD–CI, optimal performance was achieved with an L1/L2 ratio of 0.05 and *C* = 0.46 (accuracy = 0.67 [0.16]; balanced accuracy = 0.64 [0.18]; ROC-AUC = 0.56 [0.20]; PR-AUC = 0.54 [0.23]).

To evaluate model stability and reproducibility, weight profile consistency was assessed at three levels: (1) feature-weight vectors (FC weights), (2) IC-space maps derived from those weights, and (3) voxel-level spatial gradients. Across all 7 LOOCV folds, feature-weight vectors were highly correlated (PTSD + CI: 0.89 [0.07]; PTSDΔCI: 0.80 [0.06]; PTSD-CI: 0.83 [0.03]; Figs. S29–S31), indicating robust weight patterns despite modest variability in fold performance. After mapping FC weights into IC space, correlations remained high (PTSD + CI: 0.93 [0.05]; PTSDΔCI: 0.81 [0.08]; PTSD–CI: 0.81 [0.08]; Figs. S32–S34). Mapping further into voxel space preserved these patterns (PTSD + CI: 0.93 [0.05]; PTSDΔCI: 0.80 [0.08]; PTSD–CI: 0.74 [0.14]; Figs. S35–S37). Overall, these results demonstrate strong within-model stability across folds and reproducible identification of discriminative network-level patterns. Consistent PTSD-related CI signatures were observed across the PTSD + CI and PTSDΔCI models (voxel-space correlation *r* = 0.83), but not in PTSD–CI (*r* = –0.099), suggesting a set of FC alterations specifically associated with cognitive impairment in PTSD.

**Fig. 3:**
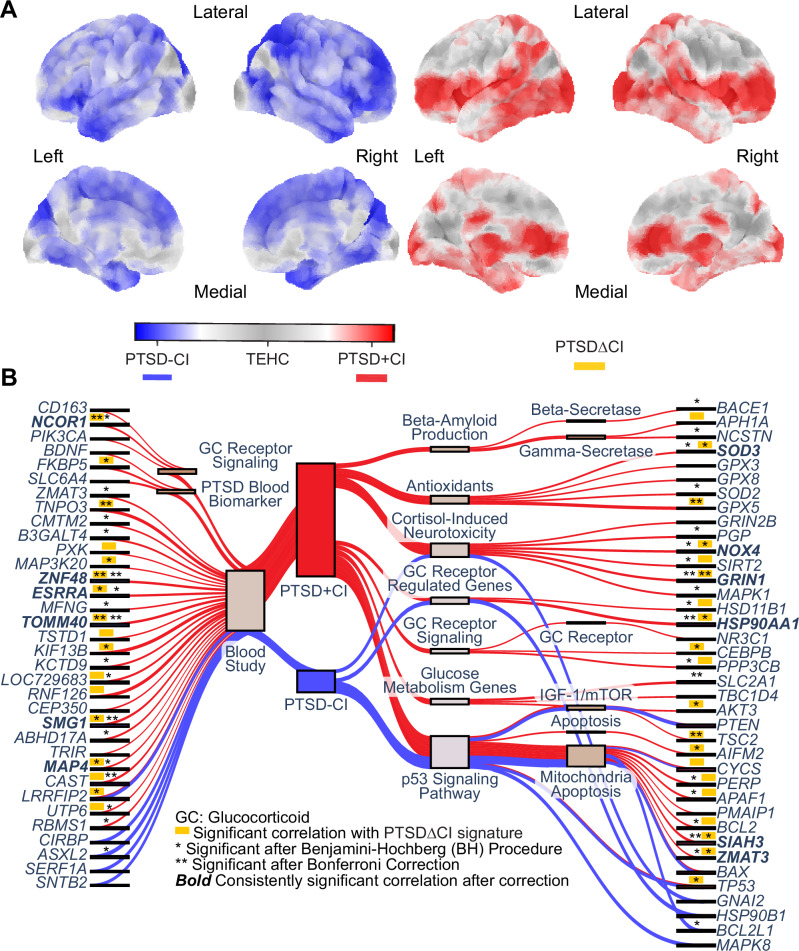
The data identified two functionally related but distinct brain signatures for PTSD with and without cognitive impairment. **A** The PTSD + CI vs TEHC and PTSD-CI vs TEHC models highlighted distinct brain signatures. Pearson correlation between the two images is –0.026. **B** All signatures demonstrate significant spatial correlations with p53-induced apoptosis genes and differentially expressed genes in blood from a separate study on WTC responders. However, the PTSD + CI and PTSDΔCI signatures exclusively exhibit significant correlations with cortisol-related genes and the significant blood biomarkers identified in other studies of the WTC cohort. GRIN1, ZNF48, and TOMM40 showed consistent, highly significant correlations with PTSD + CI and PTSDΔCI signatures. *See Patients and Methods and Table S1 for specific functions for genes and references.

When incorporating class-weight balancing, the testing performance of PTSD + CI models showed a modest improvement (accuracy = 0.77 [0.07]; balanced accuracy = 0.76 [0.08]; ROC-AUC = 0.86 [0.09]; PR-AUC = 0.75 [0.22]), whereas the performance of PTSD–CI models decreased (accuracy = 0.55 [0.099]; balanced accuracy = 0.56 [0.12]; ROC-AUC = 0.55 [0.22]; PR-AUC = 0.53 [0.23]). For PTSD + CI, brain signatures with and without class balancing were highly correlated (*r* = 0.997), indicating minimal impact on feature importance. Therefore, class balancing was not applied in the final optimization of PTSD–CI models. Taken together, these analyses yielded specific brain signatures for each phenotype (Fig. 3A; PTSDΔCI in Fig. S1). At the cortical level, we observed more significant correlations (*p* < 0.05 after spatial auto-correlation correction) for genes linked to the PTSD + CI signature (63/168) and PTSDΔCI signature (58/168) than for the PTSD–CI signature (14/168) (full cortical level correlations in Table S1) (Fig. 3B).

### P53 apoptosis genes

All cohort signatures spatially correlated with p53 apoptosis genes (11/28 in PTSD + CI, 12/28 in PTSDΔCI, and 7/28 in PTSD–CI) and PTSD peripheral study [24] related genes (30/82 in PTSD + CI, 27/82 in PTSDΔCI, and 5/82 in PTSD–CI). Commonly correlated p53-related genes between PTSD + CI and PTSD-CI included *BAX*, *CYCS*, *PTEN*, and *TP53*; of these, *TP53* and *CYCS* correlated with all signatures. Both PTSD + CI and PTSDΔCI consistently correlated with *AIFM2*, *PERP, APAF-1, BCL2, SIAH3, and ZMAT3*. Notably, only PTSD + CI correlated with *BDNF*, *SLC6A4*, and *FKBP5* genes identified by the prior peripheral study [24], although PTSDΔCI replicated with just *FKBP5*.

### Glucocorticoid neurotoxicity genes

Other genes related to glucose metabolism (3/9), GC receptor (5/17), β-amyloid (3/8), antioxidants (5/11), and cortisol-induced neurotoxicity (6/13) exclusively correlated with PTSD + CI, with 12 of 22 significant correlations replicating with PTSDΔCI. Post BH correction, 42 of 63 genes correlated with PTSD + CI, 28 of 58 genes correlated with PTSDΔCI, and 2 of 14 with PTSD–CI survived statistical thresholding. Genes related to cortisol-induced neurotoxicity and GC receptors, except *NR3C1, GRIN2B,* and *CEBPB*, were unaffected in PTSD + CI. After Bonferroni correction (*p* < 1.5e-4), eight genes remained significant with PTSD + CI, 10 with PTSDΔCI, and one with PTSD-CI (Fig. 3B). Thirteen correlations (Fig. 3B, bolded) with both PTSD + CI and PTSDΔCI survived multiple-comparison correction. Three genes, *ZNF48* (Spearman’s R: 0.30; *p* = 9.7e-5 in PTSD + CI; Spearman’s R: 0.33; *p* = 1.8e-5 in PTSDΔCI), *TOMM40* (Spearman’s R: –0.31; *p* = 1.1e-4 in PTSD + CI; Spearman’s R: –0.34; *p* = 2.7e-5 in PTSDΔCI), and *GRIN1* (Spearman’s R: 0.29; *p* = 1.9e-5 in PTSD + CI; Spearman’s R: 0.33; *p* = 9.6e-7 in PTSDΔCI), shown consistent, highly significant correlations that survived Bonferroni correction in both PTSD + CI and PTSDΔCI.

### Pathways identified using structural equational modeling (SEM)

**Fig. 4:**
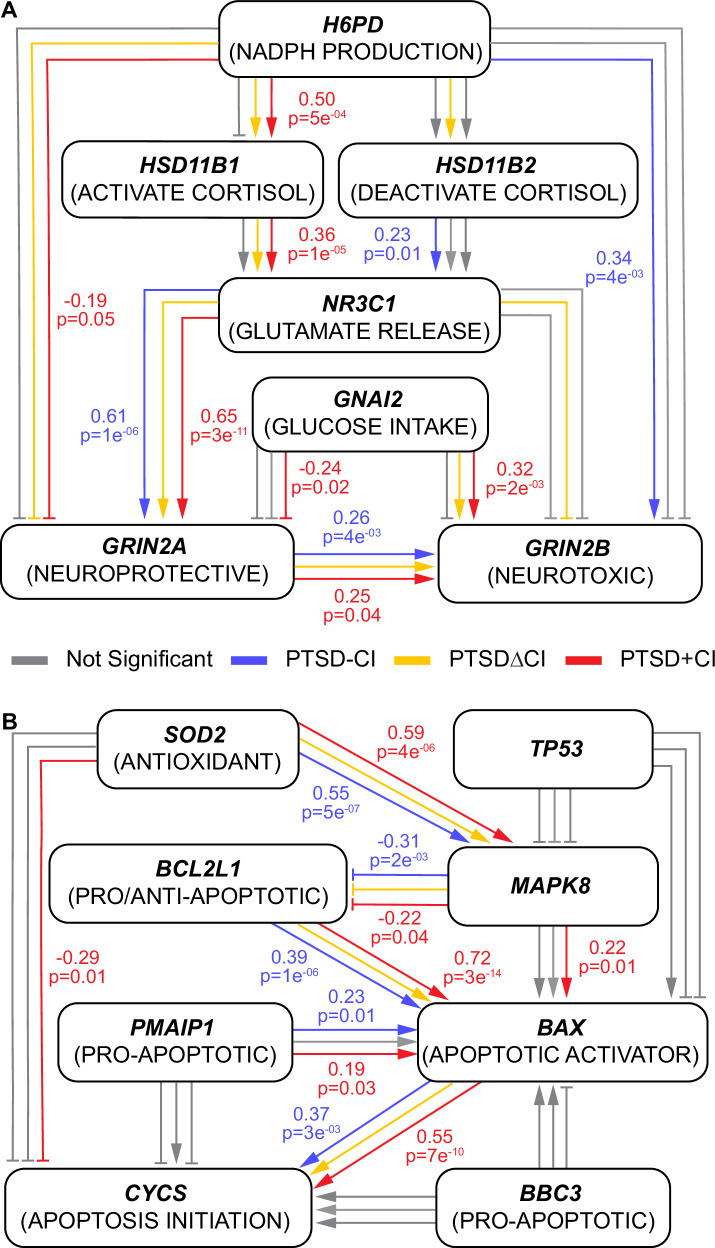
SEM inferred a cortisol-induced neurotoxicity cascade exclusively in PTSD + CI and PTSDΔCI, while a ROS/JNK/p53 apoptosis cascade in all phenotypes. **A** Qualitative shifts in cortisol-induced neurotoxicity between PTSD + CI and PTSD–CI were shown and validated in PTSDΔCI. The NMDAR2A (*GRIN2A*) neuroprotection is negatively associated with cortisol activation in PTSD + CI and PTSDΔCI, while not in PTSD–CI. **B** Quantitative alterations in p53-induced apoptosis among PTSDΔCI, PTSD + CI, and PTSD–CI were shown. A pro-apoptotic trend is shown in all signatures.

SEM identified a *ROS/JNK/p53 apoptosis* pathway for all signatures, and a *cortisol-induced neurotoxicity* pathway just for PTSD + CI and PTSDΔCI (Fig. 4). Permuted models yielded two SEM models with reasonable fit across PTSD + CI and PTSD–CI. The cortisol-induced neurotoxicity pathway (Fig. 4A) showed an excellent fit for PTSD + CI (CFI: 0.98, TLI: 0.96, & RMSEA: 0.046) and a moderate fit for PTSD–CI (CFI: 0.94, TLI: 0.89, & RMSEA: 0.051). The ROS/JNK/p53 pathway (Fig. 4B) showed a moderate fit for PTSD + CI (CFI: 0.96, TLI: 0.93, & RMSEA: 0.061) and an excellent fit for PTSD–CI (CFI: 0.98, TLI: 0.97, & RMSEA: 0.034). The cortisol-induced neurotoxicity pathway did not reliably fit the PTSDΔCI signature (CFI: 0.89, TLI: 0.80, & RMSEA: 0.11). In contrast, the ROS/JNK/p53 pathway fit the PTSDΔCI signature exceptionally well (CFI:1, TLI: 1, & RMSEA: 0). CFI and TLI were equal to 1 due to the chi-square being smaller than its degree of freedom (20). The PTSDΔCI SEM results were treated as complementary to support findings from PTSD + CI and PTSD-CI models, whose performances were optimized over permuted models.

For the cortisol-induced neurotoxicity pathway, the addition of CI to PTSD caused five of 11 connections to shift from not significant to significant (*p* < 0.05). Six out of seven connections in the PTSD + CI model were replicated in the PTSDΔCI model (Fig. S3). The two shared connections are quantitatively close between PTSD + CI and PTSD-CI. For the ROS/JNK/p53 pathway, the addition of CI to PTSD caused two of 12 connections to shift from not significant to significant. Five connections were consistently significant between PTSD + CI and PTSD-CI, with stronger positive associations observed from *SOD2* to *MAPK8* to *BCL2L1* to *BAX* to *CYCS* in PTSD + CI. These connections were replicated in the PTSDΔCI model with an association strength between PTSD + CI and PTSD-CI (see Fig. S2 for specific values).

Due to the limited sample size and the high dimensionality of features, we used several strategies to address overfitting. First, voxel-level data were reduced into IC space to constrain the final functional connectivity FC feature space. Second, models were trained using 7-fold LOOCV, ensuring that a reasonable number of samples were available for testing while retaining most samples for training. Third, the elastic net hyperparameters (L1/L2 ratio and C) were tuned across the folds and optimized based on mean testing accuracy. Fourth, model weights in FC space were mapped back into IC space, and results were averaged across folds to minimize fold-specific noise. Fifth, stability and reproducibility within and between models were evaluated at multiple levels to further reduce model stochasticity and ensure only consistent, reproducible signatures were interpreted. Finally, while PTSD + CI and PTSD–CI were considered the primary models due to their stronger fit in SEM, the same analyses were repeated using the PTSDΔCI model. This latter model demonstrated better testing performance but had a smaller sample size and poorer SEM fits, allowing for a consistency evaluation of the results. For each comparison pair, optimal models were trained using elastic net regularization.

## Discussion

Although the spatial patterns derived from the elastic-net models map onto neurocircuitry implicated in PTSD and CI, these signatures should be interpreted as predictive patterns rather than definitive mechanistic biomarkers. The consistent FC features selected by the elastic-net model reflect discriminative connections among large-scale networks and, when mapped back into IC and voxel space, produce spatial gradients across the cortex. These models extract reproducible and stable signatures within the connectivity space, but they do not, on their own, establish causal circuitry or disease-defining markers. In this study, mechanistic interpretation is instead supported by independent evidence, including spatial alignment with mRNA expression gradients, and SEM grounded in established biological cascades. Thus, the machine-learning signatures highlight networks whose individual variability is systematically informative for distinguishing PTSD-related CI, and their biological relevance emerges only when considered in conjunction with convergent findings from PTSD and CI neuroimaging research. The stability analyses across folds and across phenotype definitions further indicate that the identified patterns reflect consistent network-level alterations rather than fold-specific fluctuations.

Machine-learning-derived brain signatures suggest that PTSD–CI implicates primarily the *prefrontal-limbic circuit*, with the *amygdala* and *orbitofrontal cortex* providing excitatory and inhibitory components, respectively, for the control system regulating emotion. In contrast, PTSD + CI implicates primarily the *corticostriatal circuit*, with the *head of the caudate nucleus* and *insula* associated with encoding of positive and negative feedback, respectively, for the control system regulating learning. In the PTSD–CI signature, the temporal pole, orbitofrontal cortex [57], right superior frontal gyrus [58, 59], parahippocampus [60, 61], and amygdala [59, 62, 63] show altered activation and connectivity in PTSD. Regions highlighted in the PTSD + CI signature, excluding subcortical regions, show altered activity in PTSD [59, 62–68], including frontal pole activity [63], amygdala-postcentral connectivity [64], and superior/middle frontal gyrus activation [59], all predictive of PTSD severity. The insula [69, 70], precuneus [71], and postcentral [72] exhibit altered activities in CI studies.

Individuals with PTSD who went on to develop early CI showed biomarker patterns consistent with cortisol-induced neurotoxicity through modulation of NMDAR activity. Cortisol levels are generally lower in PTSD compared to TEHC during rest periods [12, 13], but they can spike higher than TEHC levels during trauma exposure and recovery phases [11]. The severity of PTSD symptoms during trauma script exposure correlates with these elevated cortisol levels [11]. The metabolic hypothesis suggests altered GC sensitivity in PTSD [73, 74], with evidence of heightened sensitivity to GC in PTSD blood samples [75]. To counteract this hypersensitivity, up-regulation of FKBP5 in PTSD is observed, which reduces GC receptor affinity to cortisol through conformational changes in binding [24, 76]. This regulatory shift helps mitigate damage from cortisol during rest periods, but it may exacerbate the effects of elevated cortisol levels during trauma exposure, leading to allostatic load. Additionally, reducing GC receptor affinity frees up more circulating cortisol. Studies also indicate greater cognitive impairment in PTSD individuals following acute GC administration, underscoring the detrimental impact of altered GC response and sensitivity [77].

Under dysregulated cortisol responses, an excess amount of circulating cortisol damages neurons via cortisol inhibiting the neuroprotective effects of NMDAR2A (*GRIN2A*) [38]. Under stress conditions, activities of glutamate receptors NMDARs in the prefrontal cortex (PFC) [78] and extracellular glutamate levels in the hippocampus, amygdala, and PFC [79] are increased. While NMDAR is in an activated stage, the excess amount of circulating cortisol alters NMDAR function [38]. Our PTSD + CI and PTSDΔCI models support a causal pathway that shifts NMDAR function from a dual role (both NMDAR2A and NMDAR2B (*GRIN2B*) activated) to predominantly neurotoxic. The shift is supported by positive associations between NMDAR2B and enhanced glucose uptake (*GNAI2*), and negative associations between NMDAR2A and cortisol activation and NADPH production (*H6PD*), which promotes glutamate release (*NR3C1*) through cortisol activation (via *HSD11B1*) [52].

In contrast, the PTSD–CI model shows similar positive associations from glutamate release to NMDAR2A to NMDAR2B without negative associations to NMDAR2A and positive associations to NMDAR2B from cortisol-related processes [38]. Here, enhanced glucose uptake likely promotes NMDAR2B activity, facilitated by glucose conversion to glutamate [49]. All three models feature a positive association from NMDAR2A to NMDAR2B. Such a positive association is also true in the other direction, suggesting that interactions between NMDAR2A and NMDAR2B are more complex than a linear relationship. All models show a promoting pathway from glutamate release to NMDAR function. Yet, the cortisol-induced neurotoxicity model delineates a comprehensive causal pathway of cortisol-altered glutamatergic signaling [38] that aligns with the functional signature of PTSD + CI and PTSDΔCI. However, two connections in PTSD–CI and two connections in PTSDΔCI remain unexplained by this hypothesis, potentially contributing to the weaker model fit in PTSD–CI and PTSDΔCI compared to PTSD + CI. In summary, our findings suggest that exaggerated cortisol responses promote excitotoxicity by shifting NMDAR function toward neurotoxicity. This mechanism may contribute to neuronal damage and the accelerated cognitive impairment observed in PTSD.

The ROS/JNK/p53 pathways in all groups suggest activation of apoptosis in PTSD [31], independent of cognitive decline, involving pro-apoptotic genes (*BCL2L1* and *NOXA*), an apoptotic activator (*BAX*), and apoptosis initiation (*CYCS*). These findings implicate p53-related apoptosis as a consistent feature of PTSD brain function. *TP53* connections were not significant, which may reflect its lack of specificity, as it plays a key role not only in apoptosis but also in multiple other cellular processes [80]. The specific positive association between MAPK8 and BAX and the negative association between SOD2 and CYCS in PTSD + CI are inconsistent with the main replicated apoptosis causal pathway [55]. Similar to *TP53*, the conflicting effects of *MAPK8* on apoptosis may also be due to its involvement in multiple processes [31]. The primary difference among the groups is the involvement of antioxidants in PTSD + CI, potentially explained by cortisol activation draining the NADPH pool, a significant antioxidant that regulates ROS levels and oxidative stress [81]. Together, these findings suggest that apoptosis is broadly engaged in PTSD, but the severity of apoptotic signaling increases progressively from PTSD–CI to PTSDΔCI to PTSD + CI. A more pronounced apoptosis cascade may contribute to neuronal damage, thereby linking PTSD to accelerated cognitive impairment rather than representing two independent conditions.

The cortical-level correlations between brain signatures and mRNA expression further validate that accelerated CI is associated with cortisol-induced neurotoxicity, whereas p53 apoptosis is a more general feature of PTSD. Although the correlations between *GRIN2B* and PTSD + CI and between *GRIN2A* and PTSDΔCI did not survive FDR correction, *GRIN1* is one of the three genes that show correlations with both PTSD + CI and PTSDΔCI signatures after Bonferroni correction (Fig. 3B). While the other two genes, *ZNF48* and *TOMM40*, from the previous peripheral biomarkers study [24] show a potential in bridging peripheral findings to the central nervous system, GRIN1 specifically encodes the required subunit for NMDAR, which is the central part of the cortisol-induced neurotoxicity hypothesis. The convergence of NMDAR involvement across SEM and spatial correlation analyses with both PTSD + CI and PTSDΔCI implicates involvement of excitotoxicity specific to CI in our cohort. Genes involved in GC uptake (*PGP*), GC activation (*HSD11B1*), glucose uptake (*NOX4*, *SLC2A1*) [82], and NADPH production up-regulation (*SIRT2*) are anti-correlated with the PTSD + CI and PTSDΔCI signature. These anti-correlations suggest that excess activated cortisol could impair neurons while NMDAR drives the functional signature. Among the p53-related genes, genes for major apoptosis factors (*TP53* and *CYCS*) correlate with all PTSD signatures. Additionally, antioxidants are anti-correlated with the PTSD + CI signature. Together, these results suggest dysregulation across all PTSD-related signatures, but with greater neuronal vulnerability in PTSD-related CI. The more substantial involvement of antioxidant pathways in PTSD + CI is consistent with NADPH depletion, which limits antioxidant defense and exacerbates oxidative damage.

While our findings are suggestive and promising, there are limitations in our design to be addressed by future studies. First, while our sample had unique advantages in terms of face validity and uniformity of trauma exposure, it also primarily consisted of male law enforcement officers. Therefore, the generalizability of our findings to females or other populations with lower pre-trauma baseline stress resilience remains an important direction for future research. While sex may contribute to higher PTSD onset in females [83–85], occupational demographics limited equal inclusion of both sexes in our study. The high specificity of this cohort, in terms of sex, occupation, and type of trauma exposure, likely influences the results. Therefore, replication in more diverse samples is essential to assess generalizability across broader PTSD populations. Second, the mRNA expression maps used in the study were obtained from the postmortem brains of six healthy donors, while the brain signatures were extracted from a PTSD cohort. Both postmortem interval [86–88] and PTSD status [24] are known to influence mRNA expression levels. Consequently, our approach does not provide individual-level resolution, although it identifies potential molecular targets for future personalized therapeutic strategies. Third, our unique sample was also small. While we optimized our pipeline to minimize overfitting and our findings replicate across several different statistical models, findings unique to a single model should be interpreted cautiously, and larger samples will be important for consolidation and validation. Fourth, we investigated cortisol-related neurotoxicity and p53 apoptosis, as well as PTSD with and without accelerated CI independently; however, these pathways and phenotypes are unlikely to be fully independent, and their causal relationships remain unclear. While our data suggest distinct cortisol-related neurotoxicity and overlapping p53-related apoptosis functional signatures, longitudinal studies with targeted interventions will be needed to disentangle their causal interplay.

Our findings suggest several new directions for future research. A first area leverages a framework that noninvasively links peripheral-to-central molecular systems, whose convergence can be used to identify more robust, systemically involved biomarkers. A good example of this would be *ZNF48* and *TOMM40*, which were the only genes to show this convergence for PTSD and related cognitive impairment. Second, our results suggest interactions between p53 apoptosis and cortisol-induced neurotoxicity. Future longitudinal studies integrating transcriptomics and glutamate (neurotransmitter to NMDAR) measures could help disentangle these biological processes in how they shape clinical trajectories over time. Third, our findings may identify biomarkers (*ZNF48, TOMM40*, and *GRIN1*) for testing (e.g., glutamate levels measured by magnetic resonance spectroscopy, cortisol regulation) as targets for early detection, intervention, and clinical management. In addition, strategies such as beta-hydroxybutyrate supplementation to counteract glucocorticoid-induced glucose hypometabolism could mitigate brain damage [20].

A key strength of this work was access to a rare, well-characterized cohort of WTC responders, which allowed us to distinguish PTSD with and without cognitive impairment in the absence of confounding factors such as head injury or chronic trauma exposure. This design provides an unusually well-controlled window into PTSD-related mechanisms and their links to cognitive decline. Together, the integration of a uniquely well-controlled cohort and multimodal, noninvasive integration at the mechanistic and biomarker scales provides a data-driven foundation for prospective, preventively oriented studies for how stress affects brain aging and associated cognitive decline.

## Supplementary information

Supplementary Figures

Supplementary Table

## Data Availability

Upon publication, all code/processing pipelines will be made available on www.lcneuro/tools. Data may be made available upon request.
