# Supplementary material for: Mechanistic signatures of comorbid PTSD with cognitive impairment implicate cortisol-induced neural toxicity
Source: Neuropsychopharmacology. 2026 Feb 12;51(7):1325–34. doi: 10.1038/s41386-026-02358-6 (PMC13213041; doi:10.1038/s41386-026-02358-6)
Supplement: Supplementary file 1 — Supplementary Figures [file 41386_2026_2358_MOESM1_ESM.pdf]

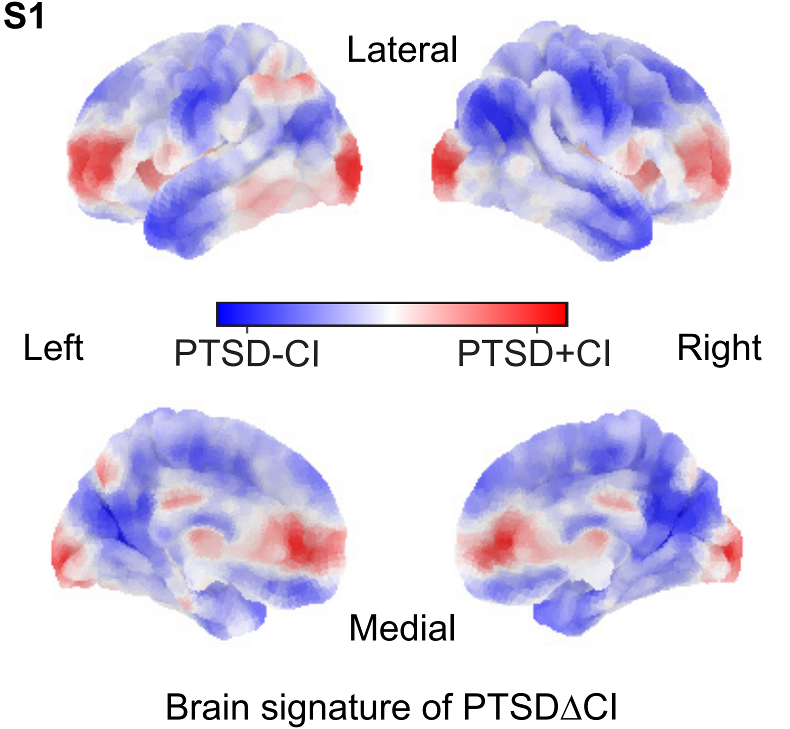
S1
Lateral
Left
PTSD-CI
PTSD+CI
Right
Medial
Brain signature of PTSDΔCI

S2

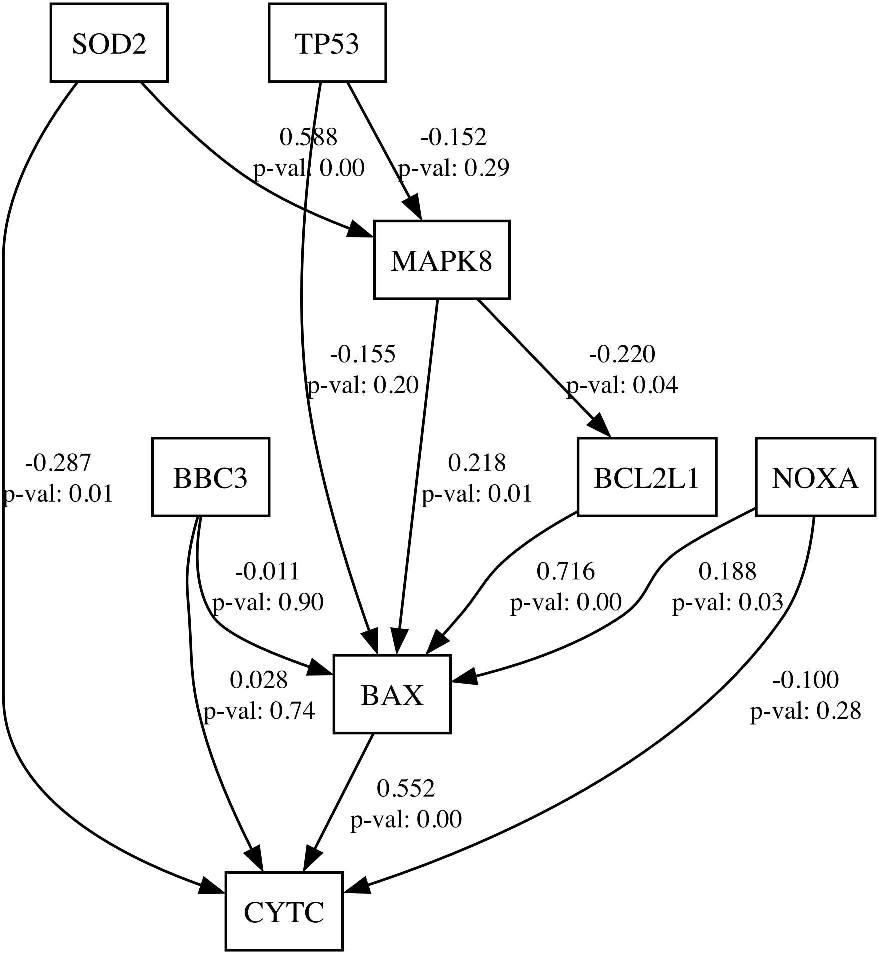

p53 apoptosis SEM fit of PTSDΔCI

S3

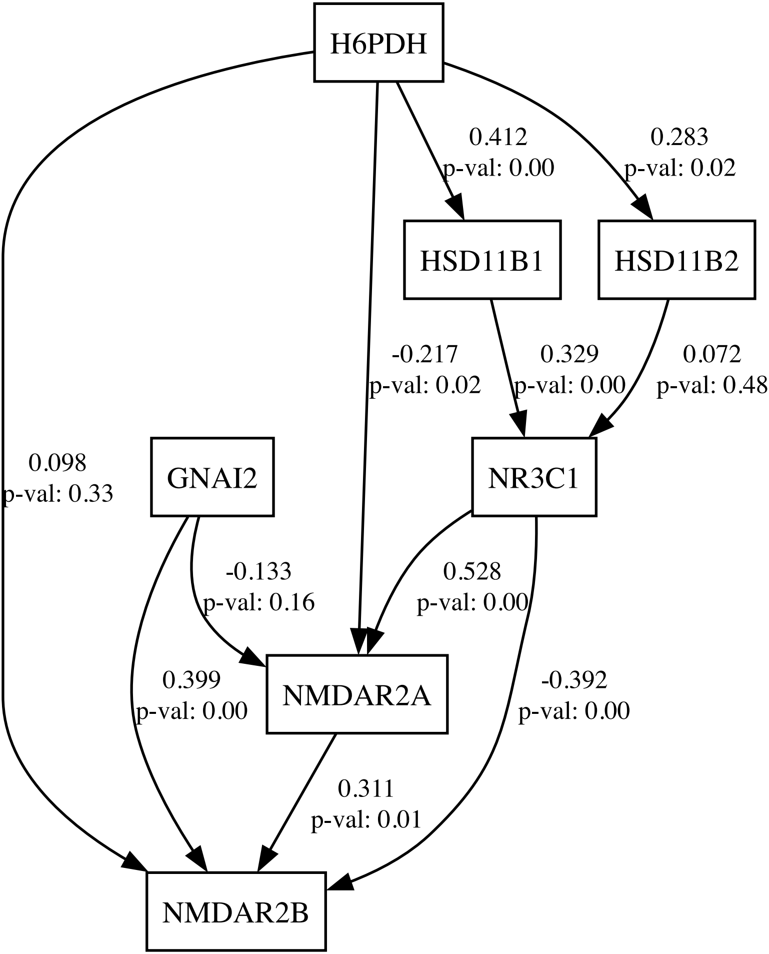

Cortisol-induced neurotoxicity SEM fit of PTSDΔCI

S4: IC1

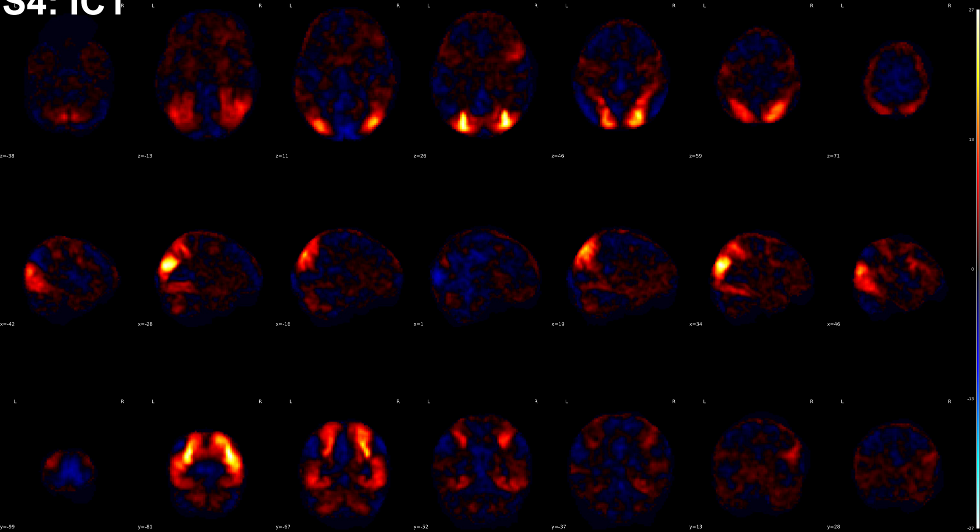
L
R
z=-38
z=-13
z=11
z=26
z=46
z=59
z=71
x=-42
x=-28
x=-16
x=1
x=19
x=34
x=46
y=-99
y=-81
y=-67
y=-52
y=-37
y=13
y=28
27
13
0
-13
-27

S5: IC2

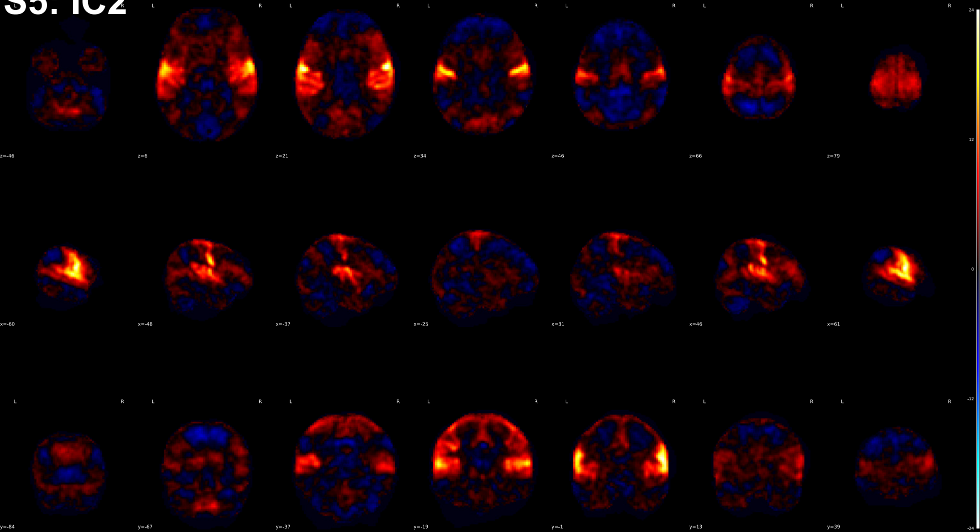

L
R
z=-46
z=6
z=21
z=34
z=46
z=66
z=79
x=-60
x=-48
x=-37
x=-25
x=31
x=46
x=61
y=-84
y=-67
y=-37
y=-19
y=-1
y=13
y=39
24
12
0
-12
-24

S6: IC3

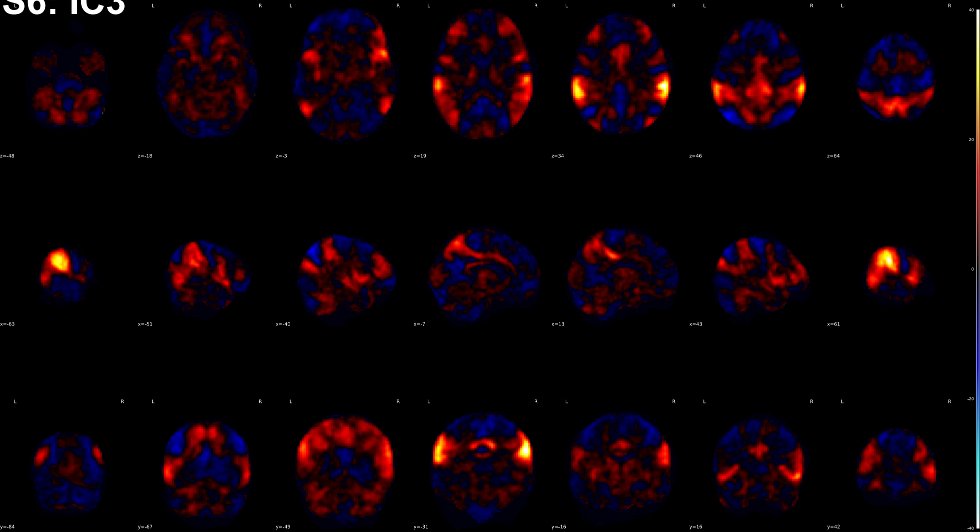

L
R
z=-48
z=-18
z=-3
z=19
z=34
z=46
z=64
x=-63
x=-51
x=-40
x=-7
x=13
x=43
x=61
y=-84
y=-67
y=-49
y=-31
y=-16
y=16
y=42
40
20
0
-20
-40

S7: IC4

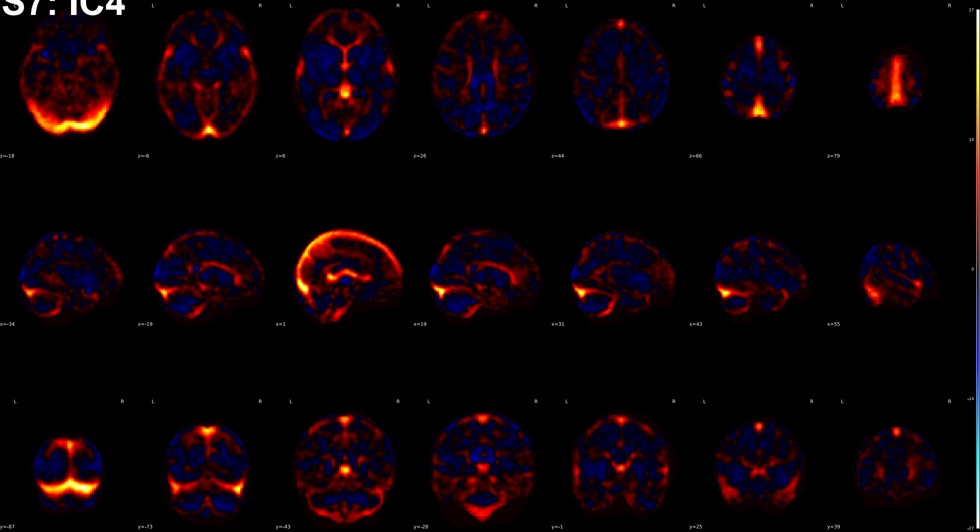

L
R
z=-18
z=-6
z=6
z=26
z=44
z=66
z=79
x=-34
x=-19
x=1
x=19
x=31
x=43
x=55
y=-87
y=-73
y=-43
y=-28
y=-1
y=25
y=39
27
14
0
-14
-27

S8: IC5

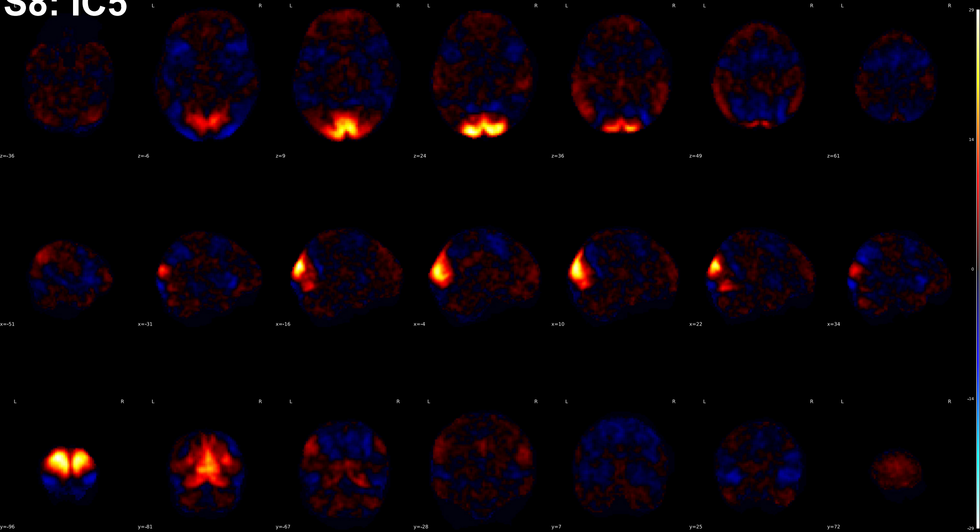
L
R
z=-36
z=-6
z=9
z=24
z=36
z=49
z=61
x=-51
x=-31
x=-16
x=-4
x=10
x=22
x=34
y=-96
y=-81
y=-67
y=-28
y=7
y=25
y=72
29
14
0
-14
-29

S9: IC6

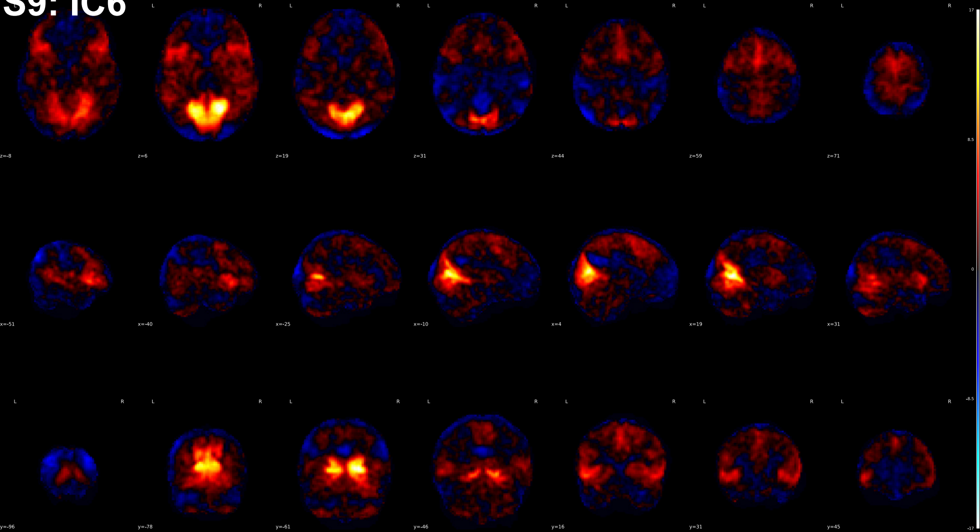

L
R
z=-8
z=6
z=19
z=31
z=44
z=59
z=71
x=-51
x=-40
x=-25
x=-10
x=4
x=19
x=31
y=-96
y=-78
y=-61
y=-46
y=16
y=31
y=45
17
8.5
0
-8.5
-17

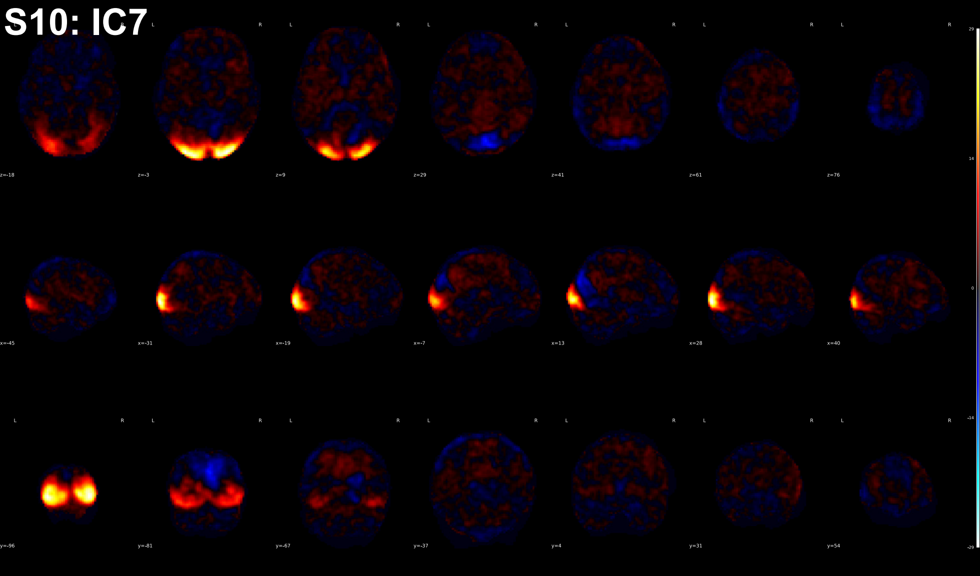
S10: IC7
z=-18
z=-3
z=9
z=29
z=41
z=61
z=76
x=-45
x=-31
x=-19
x=-7
x=13
x=28
x=40
y=-96
y=-81
y=-67
y=-37
y=4
y=31
y=54
29
14
0
-14
-29

S11: IC8

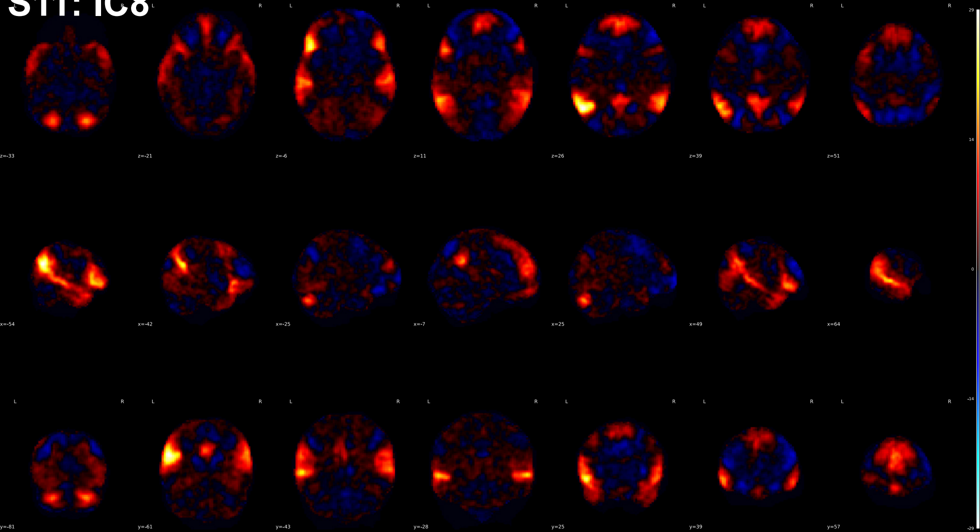
z=-33
z=-21
z=-6
z=11
z=26
z=39
z=51
x=-54
x=-42
x=-25
x=-7
x=25
x=49
x=64
y=-81
y=-61
y=-43
y=-28
y=25
y=39
y=57
29
14
0
-14
-29

S12: IC9

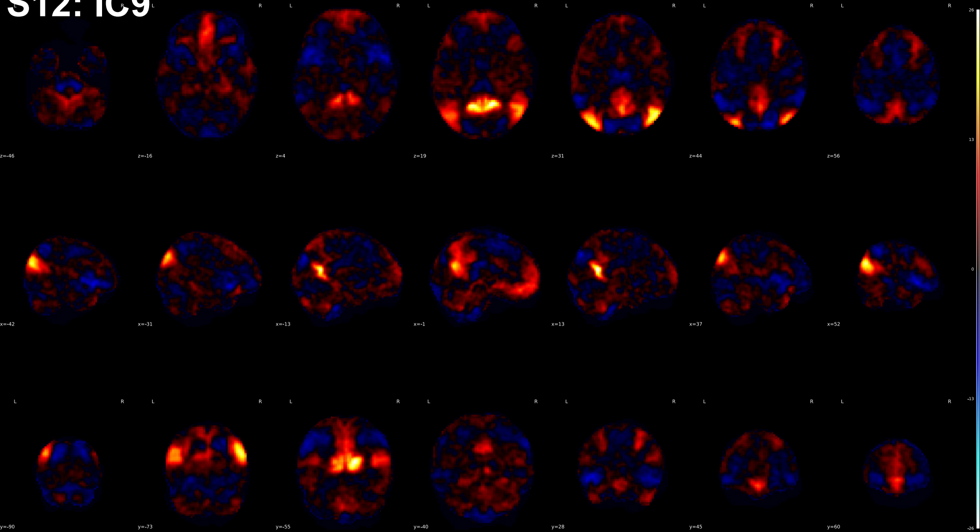

L
R
z=-46
z=-16
z=4
z=19
z=31
z=44
z=56
x=-42
x=-31
x=-13
x=-1
x=13
x=37
x=52
y=-90
y=-73
y=-55
y=-40
y=28
y=45
y=60
26
13
0
-13
-26

S13: IC10

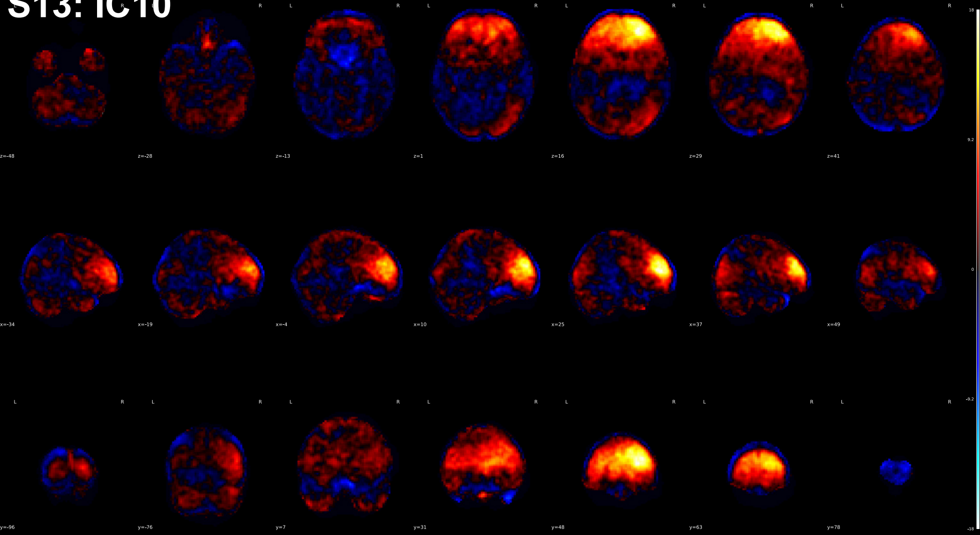

L
R
z=-48
z=-28
z=-13
z=1
z=16
z=29
z=41
x=-34
x=-19
x=-4
x=10
x=25
x=37
x=49
y=-96
y=-76
y=7
y=31
y=48
y=63
y=78
18
9.2
0
-9.2
-18

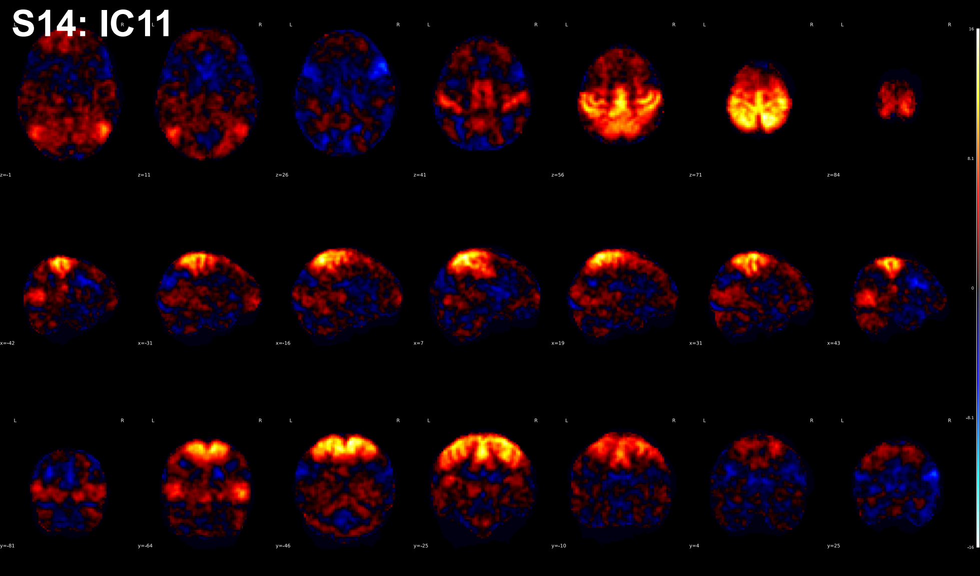
S14: IC11
L
R
z=-1
z=11
z=26
z=41
z=56
z=71
z=84
x=-42
x=-31
x=-16
x=7
x=19
x=31
x=43
y=-81
y=-64
y=-46
y=-25
y=-10
y=4
y=25
16
8.1
0
-8.1
-16

S15: IC12

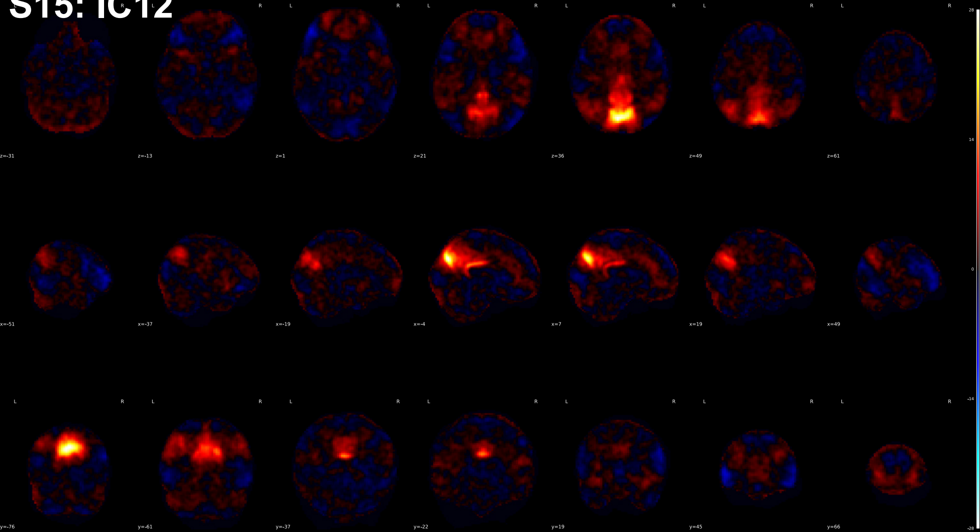

z=-31
z=-13
z=1
z=21
z=36
z=49
z=61
x=-51
x=-37
x=-19
x=-4
x=7
x=19
x=49
y=-76
y=-61
y=-37
y=-22
y=19
y=45
y=66
28
14
0
-14
-28

S16: IC13

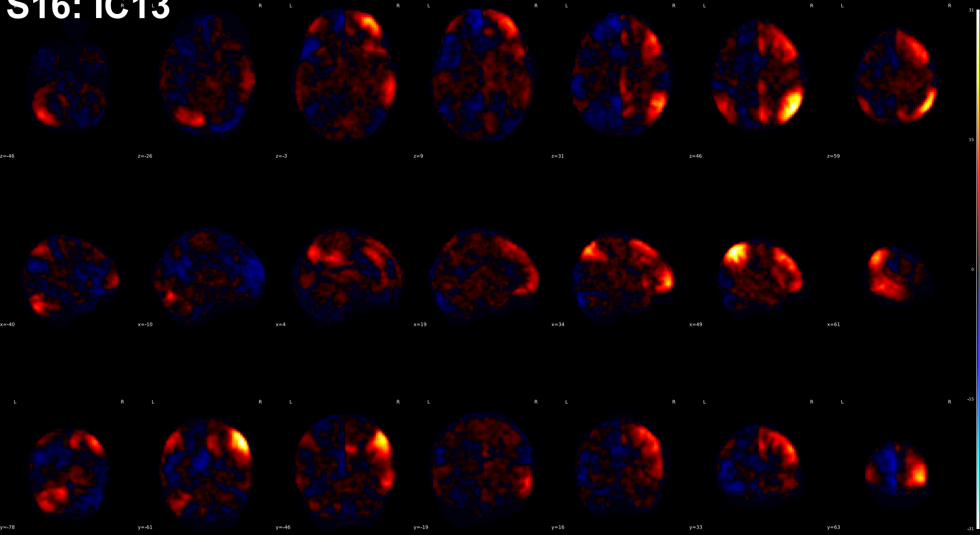

L
R
z=-46
z=-26
z=-3
z=9
z=31
z=46
z=59
x=-40
x=-10
x=4
x=19
x=34
x=49
x=61
y=-78
y=-61
y=-46
y=-19
y=16
y=33
y=63
31
15
0
-15
-31

S17: IC14

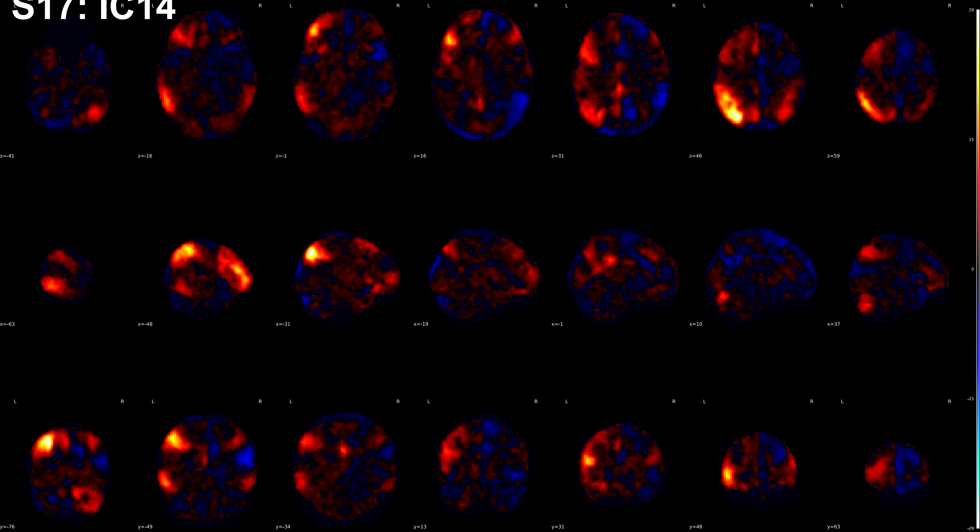
z=-41
z=-16
z=-1
z=16
z=31
z=46
z=59
x=-63
x=-48
x=-31
x=-19
x=-1
x=10
x=37
y=-76
y=-49
y=-34
y=13
y=31
y=48
y=63
29
15
0
-15
-29

S18: IC15

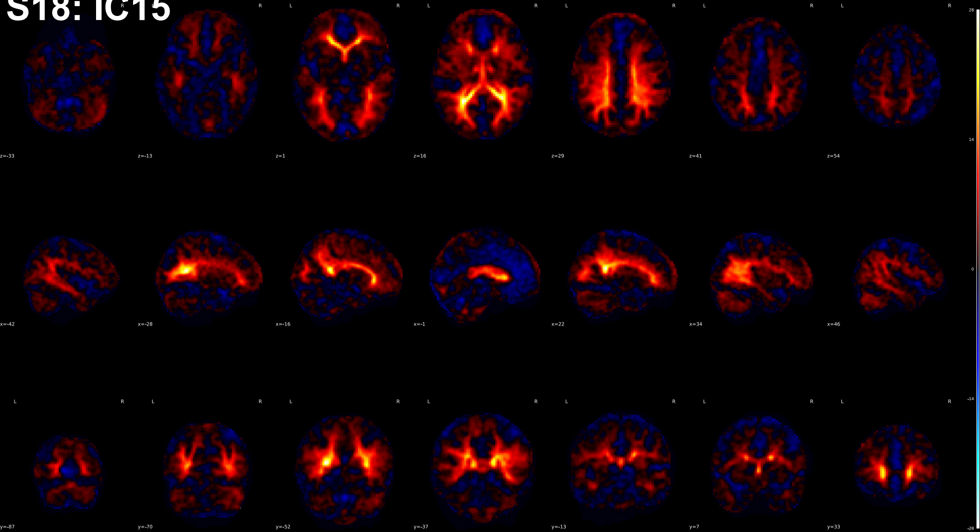

z=-33
z=-13
z=1
z=16
z=29
z=41
z=54
x=-42
x=-28
x=-16
x=-1
x=22
x=34
x=46
y=-87
y=-70
y=-52
y=-37
y=-13
y=7
y=33
28
14
0
-14
-28

S19: IC16

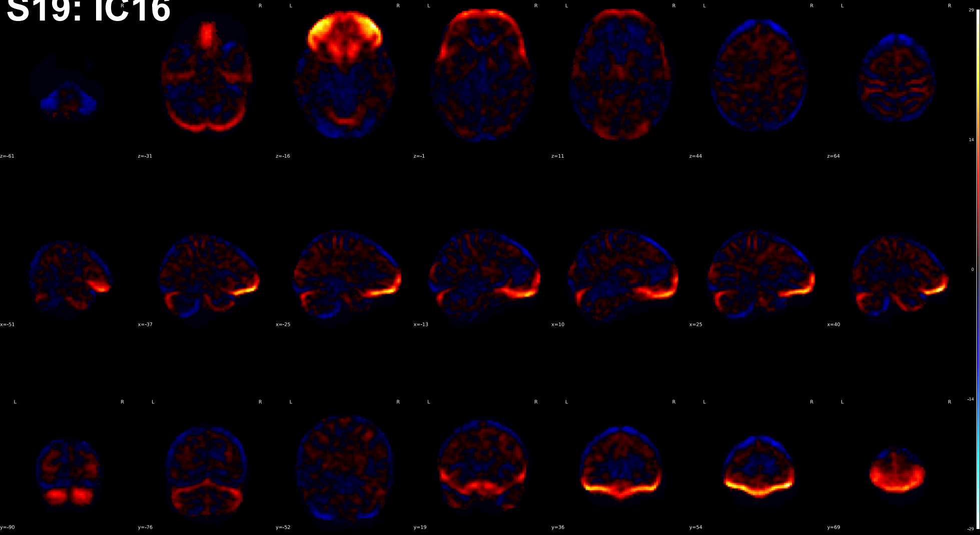

z=-61
z=-31
z=-16
z=-1
z=11
z=44
z=64
x=-51
x=-37
x=-25
x=-13
x=10
x=25
x=40
y=-90
y=-76
y=-52
y=19
y=36
y=54
y=69
29
14
0
-14
-29

S20: IC17

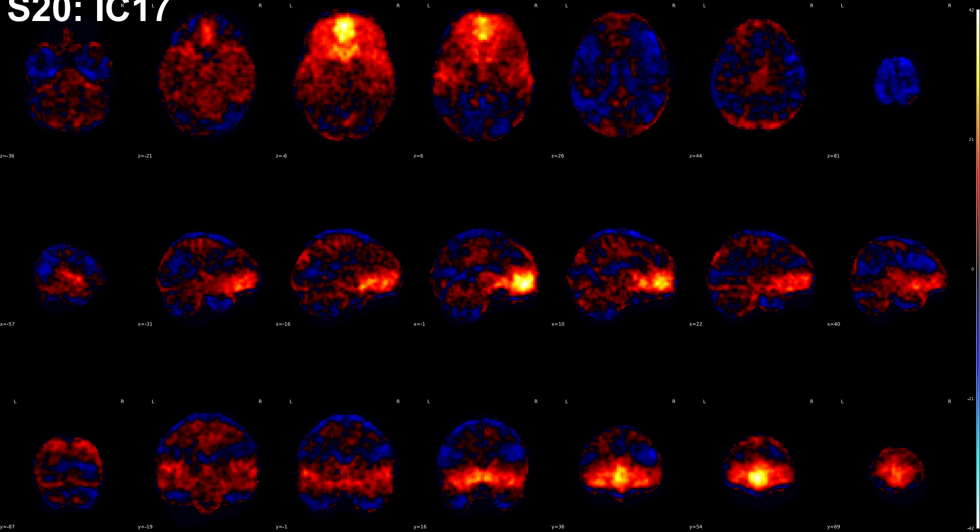
L
R
z=-36
z=-21
z=-6
z=6
z=26
z=44
z=81
x=-57
x=-31
x=-16
x=-1
x=10
x=22
x=40
y=-87
y=-19
y=-1
y=16
y=36
y=54
y=69
42
21
0
-21
-42

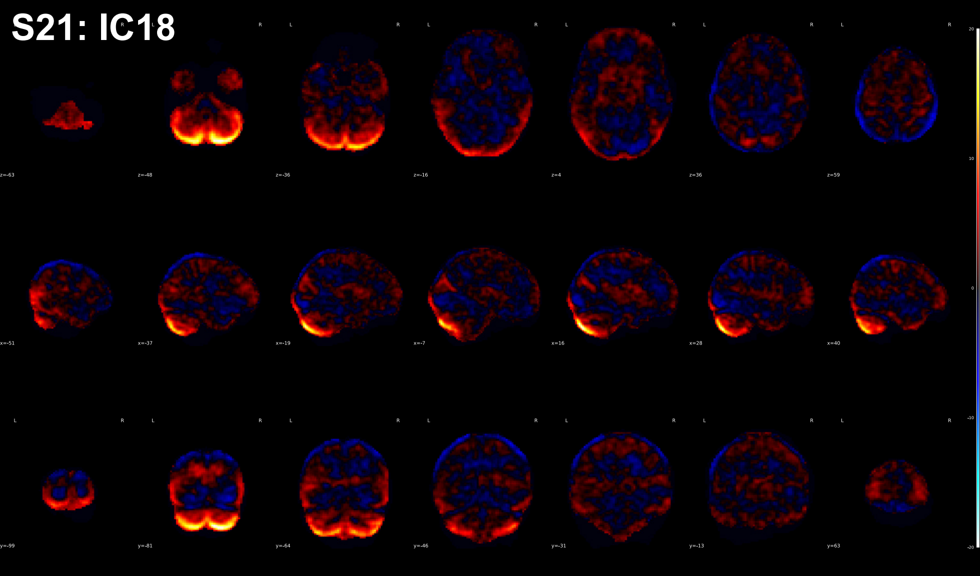

S21: IC18
z=-63
z=-48
z=-36
z=-16
z=4
z=36
z=59
x=-51
x=-37
x=-19
x=-7
x=16
x=28
x=40
y=-99
y=-81
y=-64
y=-46
y=-31
y=-13
y=63
20
10
0
-10
-20

S22: IC19

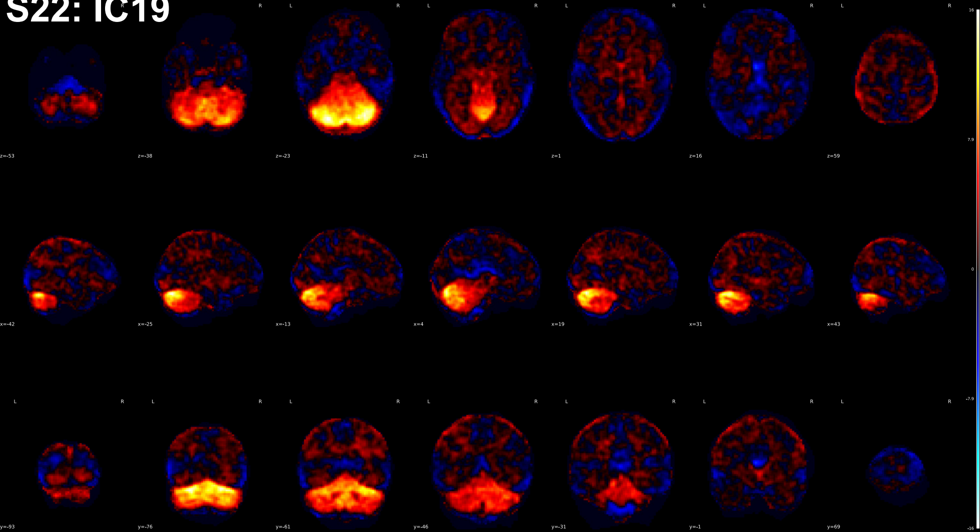
L
R
z=-53
z=-38
z=-23
z=-11
z=1
z=16
z=59
x=-42
x=-25
x=-13
x=4
x=19
x=31
x=43
y=-93
y=-76
y=-61
y=-46
y=-31
y=-1
y=69
16
7.9
0
-7.9
-16

S23: IC20

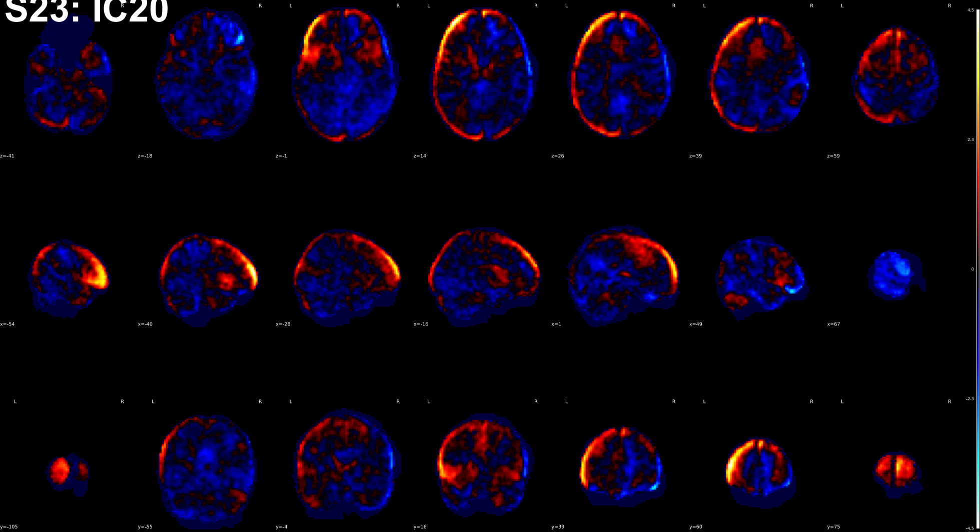

L
R
z=-41
z=-18
z=-1
z=14
z=26
z=39
z=59
x=-54
x=-40
x=-28
x=-16
x=1
x=49
x=67
y=-105
y=-55
y=-4
y=16
y=39
y=60
y=75
4.5
2.3
0
-2.3
-4.5

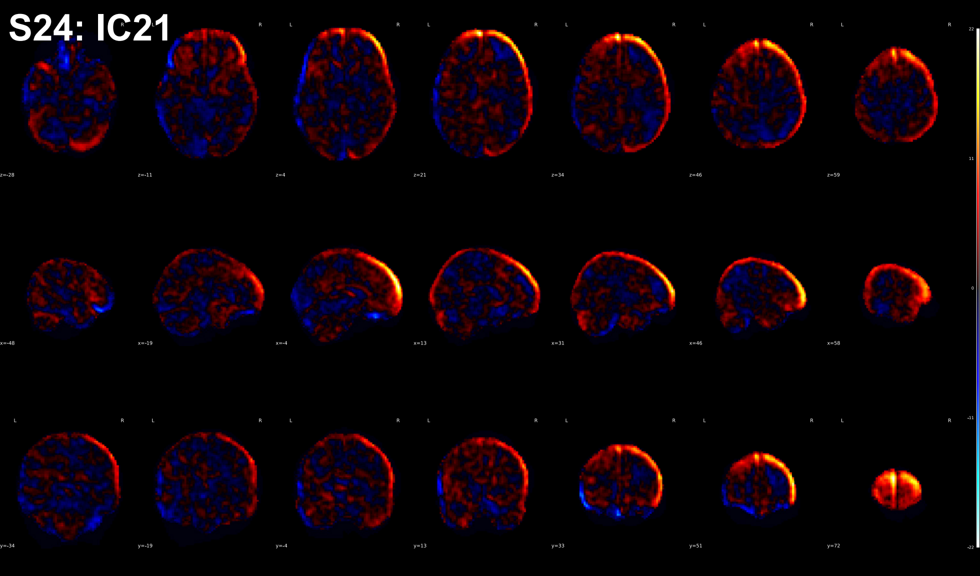
S24: IC21
z=-28
z=-11
z=4
z=21
z=34
z=46
z=59
x=-48
x=-19
x=-4
x=13
x=31
x=46
x=58
y=-34
y=-19
y=-4
y=13
y=33
y=51
y=72
22
11
0
-11
-22

S25: IC22

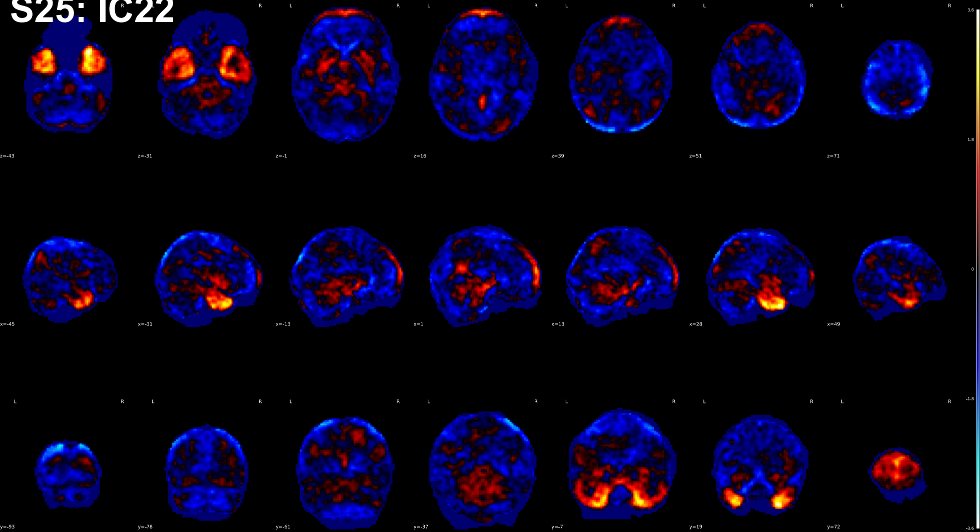
z=-43
z=-31
z=-1
z=16
z=39
z=51
z=71
x=-45
x=-31
x=-13
x=1
x=13
x=28
x=49
y=-93
y=-78
y=-61
y=-37
y=-7
y=19
y=72
3.6
1.8
0
-1.8
-3.6

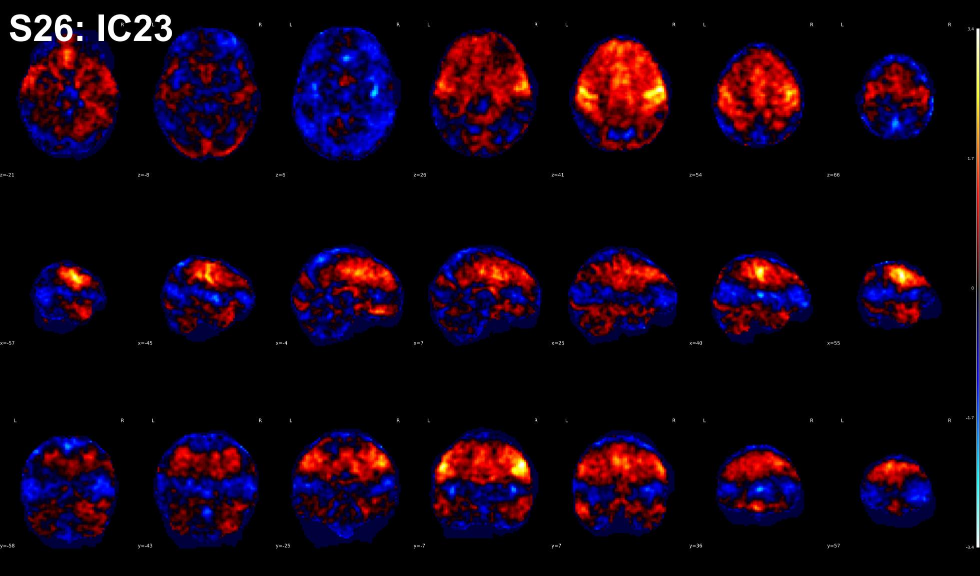
S26: IC23
z=-21
z=-8
z=6
z=26
z=41
z=54
z=66
x=-57
x=-45
x=-4
x=7
x=25
x=40
x=55
y=-58
y=-43
y=-25
y=-7
y=7
y=36
y=57
L
R
3.4
1.7
0
-1.7
-3.4

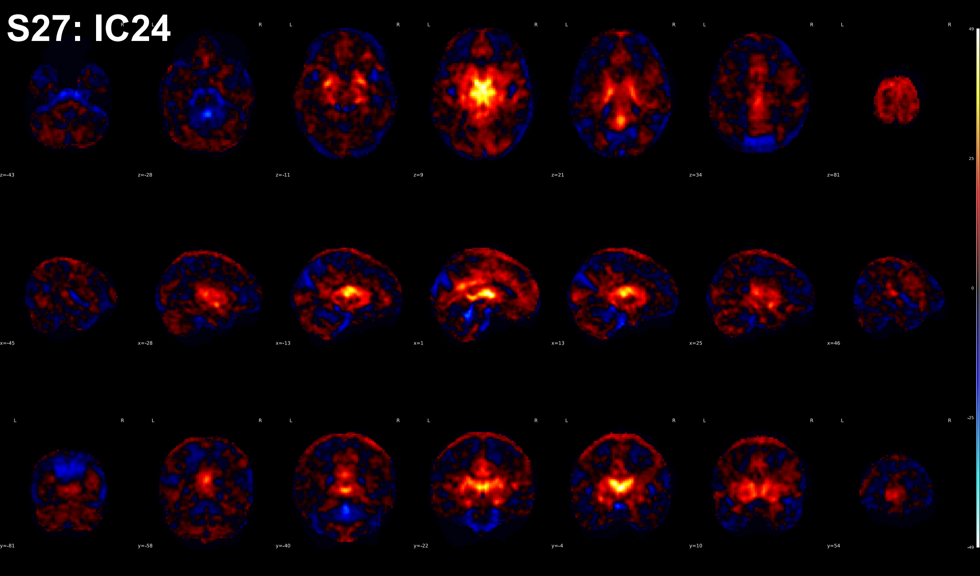
S27: IC24
z=-43
z=-28
z=-11
z=9
z=21
z=34
z=81
x=-45
x=-28
x=-13
x=1
x=13
x=25
x=46
y=-81
y=-58
y=-40
y=-22
y=-4
y=10
y=54
49
25
0
-25
-49

S28: IC25

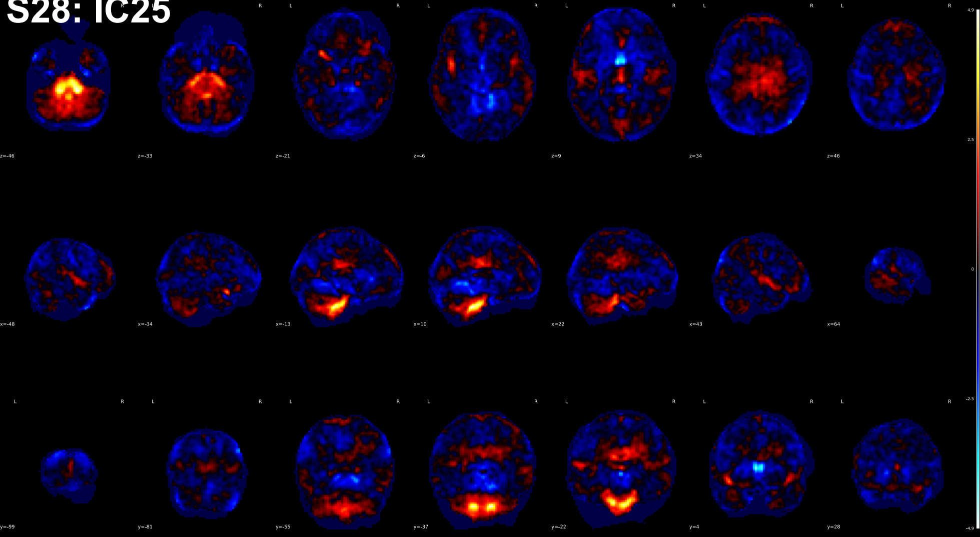

z=-46
z=-33
z=-21
z=-6
z=9
z=34
z=46
x=-48
x=-34
x=-13
x=10
x=22
x=43
x=64
y=-99
y=-81
y=-55
y=-37
y=-22
y=4
y=28
L
R
4.9
2.5
0
-2.5
-4.9

S29

PTSDΔCI

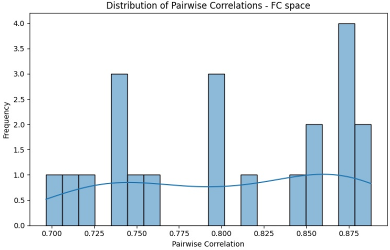

Distribution of Pairwise Correlations - FC space
Frequency
Pairwise Correlation
0.0
0.5
1.0
1.5
2.0
2.5
3.0
3.5
4.0
0.700
0.725
0.750
0.775
0.800
0.825
0.850
0.875

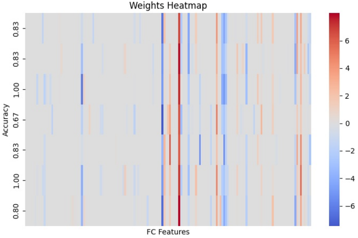

Weights Heatmap
Accuracy
0.83
0.83
1.00
0.67
0.83
1.00
0.80
FC Features
6
4
2
0
−2
−4
−6

S30

PTSD+CI

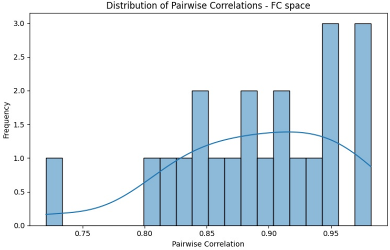

Distribution of Pairwise Correlations - FC space
Frequency
Pairwise Correlation
0.0
0.5
1.0
1.5
2.0
2.5
3.0
0.75
0.80
0.85
0.90
0.95

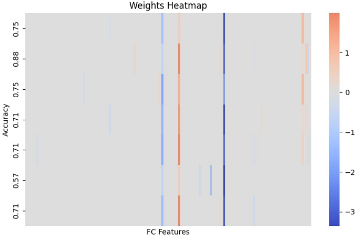

Weights Heatmap
Accuracy
FC Features
0.75
0.88
0.75
0.71
0.71
0.57
0.71
1
0
−1
−2
−3

S31

PTSD-CI

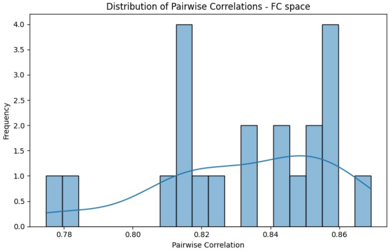
Distribution of Pairwise Correlations - FC space
Frequency
Pairwise Correlation
4.0
3.5
3.0
2.5
2.0
1.5
1.0
0.5
0.0
0.78
0.80
0.82
0.84
0.86

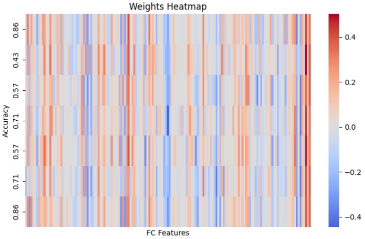
Weights Heatmap
Accuracy
FC Features
0.86
0.43
0.57
0.71
0.57
0.71
0.86
0.4
0.2
0.0
−0.2
−0.4

S32

PTSDΔCI

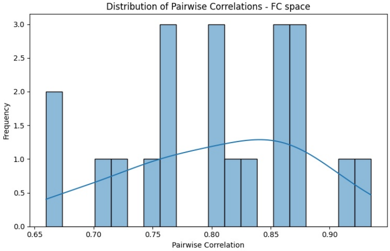
Distribution of Pairwise Correlations - FC space
Frequency
Pairwise Correlation
0.0
0.5
1.0
1.5
2.0
2.5
3.0
0.65
0.70
0.75
0.80
0.85
0.90

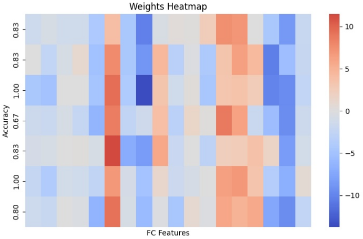
Weights Heatmap
Accuracy
FC Features
0.83
0.83
1.00
0.67
0.83
1.00
0.80
10
5
0
−5
−10

S33

PTSD+CI

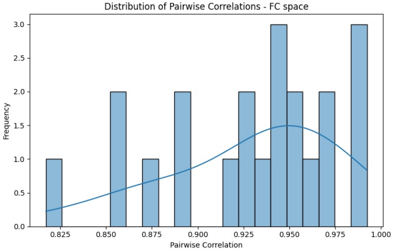
Distribution of Pairwise Correlations - FC space
Frequency
Pairwise Correlation
0.0
0.5
1.0
1.5
2.0
2.5
3.0
0.825
0.850
0.875
0.900
0.925
0.950
0.975
1.000

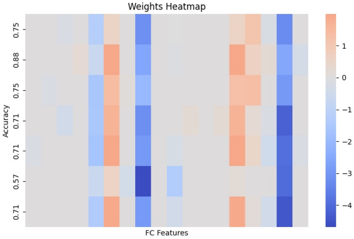
Weights Heatmap
Accuracy
0.75
0.88
0.75
0.71
0.71
0.57
0.71
FC Features
1
0
−1
−2
−3
−4

S34

PTSD-CI

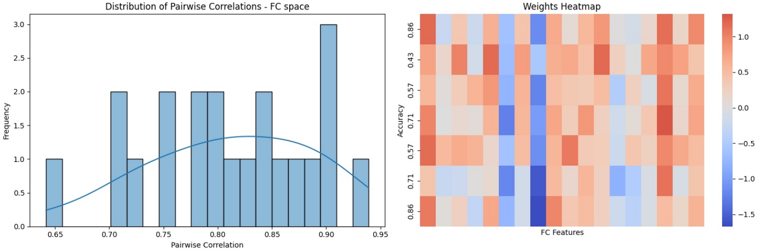
Distribution of Pairwise Correlations - FC space
Frequency
Pairwise Correlation
0.0
0.5
1.0
1.5
2.0
2.5
3.0
0.65
0.70
0.75
0.80
0.85
0.90
0.95
Weights Heatmap
Accuracy
FC Features
0.86
0.43
0.57
0.71
0.57
0.71
0.86
1.0
0.5
0.0
−0.5
−1.0
−1.5

S35

PTSDΔCI

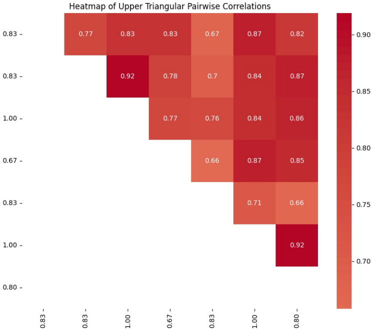

Heatmap of Upper Triangular Pairwise Correlations
0.77
0.83
0.83
0.67
0.87
0.82
0.92
0.78
0.7
0.84
0.87
0.77
0.76
0.84
0.86
0.66
0.87
0.85
0.71
0.66
0.92
0.83
0.83
1.00
0.67
0.83
1.00
0.80
0.90
0.85
0.80
0.75
0.70

S36

PTSD+CI

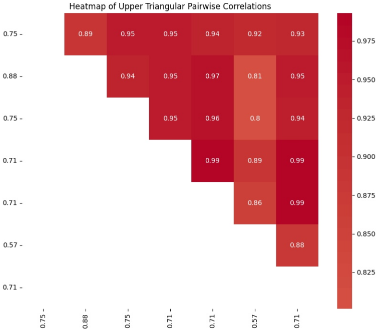
Heatmap of Upper Triangular Pairwise Correlations
0.75
0.88
0.75
0.71
0.71
0.57
0.71
0.89
0.95
0.95
0.94
0.92
0.93
0.94
0.95
0.97
0.81
0.95
0.95
0.96
0.8
0.94
0.99
0.89
0.99
0.86
0.99
0.88
0.75
0.88
0.75
0.71
0.71
0.57
0.71
0.975
0.950
0.925
0.900
0.875
0.850
0.825

S37

PTSD-CI

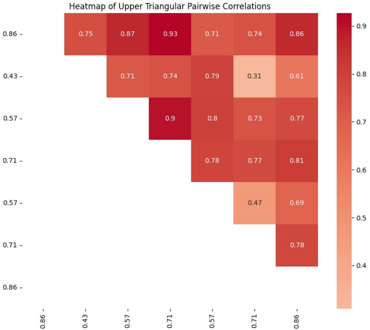
Heatmap of Upper Triangular Pairwise Correlations
0.75
0.87
0.93
0.71
0.74
0.86
0.71
0.74
0.79
0.31
0.61
0.9
0.8
0.73
0.77
0.78
0.77
0.81
0.47
0.69
0.78
0.86
0.43
0.57
0.71
0.57
0.71
0.86
0.9
0.8
0.7
0.6
0.5
0.4
